# Chemical and Light-Absorption Properties of Water-Soluble Organic Aerosols in Northern California and Photooxidant Production by Brown Carbon Components

**DOI:** 10.1021/acsearthspacechem.3c00022

**Published:** 2023-04-24

**Authors:** Wenqing Jiang, Lan Ma, Christopher Niedek, Cort Anastasio, Qi Zhang

**Affiliations:** † Department of Environmental Toxicology, University of California, 1 Shields Avenue, Davis, California 95616, United States; ‡ Agricultural and Environmental Chemistry Graduate Program, University of California, 1 Shields Avenue, Davis, California 95616, United States; § Department of Land, Air, and Water Resources, University of California, 1 Shields Avenue, Davis, California 95616, United States

**Keywords:** biomass burning organic aerosols, oxygenated organic aerosols, positive matrix factorization, hydroxyl radical, singlet oxygen, triplet excited state of organic carbon, aqueous-phase reactions, aerosol mass spectrometer

## Abstract

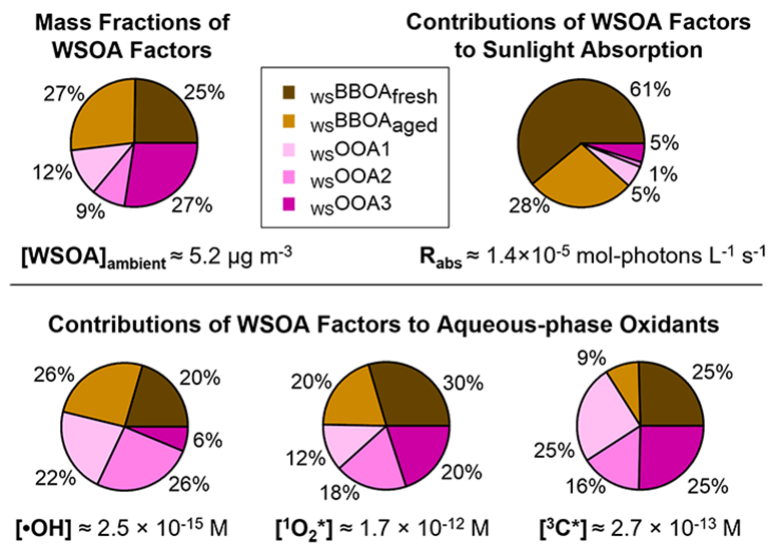

Atmospheric brown carbon (BrC) can impact the radiative balance of the earth and form photooxidants. However, the light absorption and photochemical properties of BrC from different sources remain poorly understood. To address this gap, dilute water extracts of particulate matter (PM) samples collected at Davis, CA over one year were analyzed using high resolution aerosol mass spectrometry (HR-AMS) and UV–vis spectroscopy. Positive matrix factorization (PMF) on combined AMS and UV–vis data resolved five water-soluble organic aerosol (WSOA) factors with distinct mass spectra and UV–vis spectra: a fresh and an aged water-soluble biomass burning OA (_WS_BBOA_fresh_ and _WS_BBOA_aged_) and three oxygenated OA (_WS_OOAs). _WS_BBOA_fresh_ is the most light-absorbing, with a mass absorption coefficient (MAC_365 nm_) of 1.1 m^2^ g^–1^, while the _WS_OOAs are the least (MAC_365 nm_ = 0.01–0.1 m^2^ g^–1^). These results, together with the high abundance of _WS_BBOAs (∼52% of the WSOA mass), indicate that biomass burning activities such as residential wood burning and wildfires are an important source of BrC in northern California. The concentrations of aqueous-phase photooxidants, i.e., hydroxyl radical (·OH), singlet molecular oxygen (^1^O_2_*), and oxidizing triplet excited states of organic carbon (^3^C*), were also measured in the PM extracts during illumination. Oxidant production potentials (PP_OX_) of the five WSOA factors were explored. The photoexcitation of BrC chromophores from BB emissions and in OOAs is a significant source of ^1^O_2_* and ^3^C*. By applying our PP_OX_ values to archived AMS data at dozens of sites, we found that oxygenated organic species play an important role in photooxidant formation in atmospheric waters.

## 1 Introduction

While atmospheric organic aerosols (OA) are typically considered to be light scattering,^1^ brown carbon (BrC) OA species absorb light in the visible and near-UV ranges.^2^ Unlike black carbon (BC), whose light absorption is only weakly wavelength dependent, BrC absorbs light much more efficiently at shorter wavelengths and thus has a larger absorption Ångström exponent (AAE).^2−4^ In field studies, BrC contributed up to 15% of sunlight absorption by aerosols over the UV–vis spectrum and up to 50% at shorter wavelengths.^5−7^ According to model simulations, BrC accounts for 21% of the global surface OA^8^ and has a radiative forcing in the range of 0.1–0.25 W m^–2^, approximately 25% of the BC value and enough to offset the cooling effect by nonabsorbing OA.^9,10^

By absorbing sunlight, BrC can influence photochemical reactions and oxidant concentrations in the atmosphere. BrC absorption decreases surface actinic flux, especially in the UV range, thus leading to lower gas-phase photolysis rates and lower production rates of ozone and radicals.^8,11^ On the other hand, BrC compounds are an important source of photooxidants such as oxidizing triplet excited states of organic carbon (^3^C*), singlet molecular oxygen (^1^O_2_*) and hydroxyl radical (·OH) in aerosol water and cloud/fog droplets.^12−16^ While ·OH reacts rapidly with most organics,^17^^3^C* and ^1^O_2_* can be important oxidants for electron-rich compounds, such as phenols,^18−21^ isoprene and monoterpenes,^22^ amino compounds,^23,24^ and aromatic hydrocarbons^25,26^ in the atmosphere.

The sources of BrC are complex, including direct emissions from combustion and secondary formation through reactions of biogenic and anthropogenic precursors.^3,27,28^ Global simulations estimate that burning of biomass and biofuel emits ∼3.9 and ∼3.0 Tg yr^–1^ of primary BrC, respectively, together accounting for 28% of surface BrC.^8^ Secondary BrC production is estimated at 5.7 Tg yr^–1^, contributing to 23% of surface BrC.^8^ There are various pathways that contribute to the formation of secondary BrC in both atmospheric gaseous and aqueous phases. These pathways include the formation of nitroaromatics and organonitrates through the photooxidation of aromatic hydrocarbons under high-NO_*x*_ conditions^29,30^ and the nighttime NO_3_-mediated oxidation of phenols^31^ and unsaturated heterocyclic compounds.^32^ Other pathways include reactions between ammonia or amines and carbonyls,^33−35^ the oligomerization of glyoxal and methylglyoxal during cloud processing,^36^ the aqueous formation of humic-like substances (HULIS),^37,38^ and the aqueous oxidation of phenolic compounds.^16,19,39^

The chemical composition of atmospheric BrC is complex and their optical and photochemical properties remain poorly characterized. A common approach for studying BrC is by performing solvent extractions of ambient PM samples and then measuring the UV–vis absorptivity and chemical characteristics of the PM extracts.^40−44^ Coupling aerosol mass spectrometry (AMS) with UV–vis spectrophotometry is a particularly useful method for analyzing PM extracts and providing information about the sources and processes of BrC. For example, Moschos et al. estimated the sources and light absorption properties for several major BrC components in ambient PM by applying positive matrix factorization (PMF) on the combined data set of AMS mass spectra and UV–vis spectra of the water-soluble fractions of PM from Switzerland.^40^ Furthermore, Kaur et al. recently studied the photoactivity of atmospheric BrC by determining the concentrations of major condensed-phase oxidants (i.e., ·OH, ^1^O_2_*, and ^3^C*) in dilute aqueous extracts of ambient PM and in fog waters during illumination.^12,15,45^ Bogler et al. measured steady state ^1^O_2_* concentration in PM_10_ aqueous extracts and examined the abilities of BrC components to form ^1^O_2_*.^46^

In this study, we characterized the chemical composition and optical properties of water-soluble organic aerosols (WSOA) in PM_2.5_ samples collected at Davis, a small city in northern California, over a period of one year. PMF was applied on the combined AMS and UV–vis spectral data to determine the bulk composition and mass absorption coefficient (MAC) spectra of major water-soluble BrC components. Furthermore, steady-state concentrations of ·OH, ^1^O_2_*, and ^3^C* were measured in the illuminated dilute PM extracts in our companion paper.^47,48^ Here we combine our AMS, UV–vis, and oxidant measurements to explore the relationships between WSOA composition and photoformation of aqueous oxidants. The oxidant production potentials of different WSOA components were calculated and used to estimate aqueous oxidant concentrations in clouds and fogs at dozens of sites worldwide.

## 2 Materials and Methods

### 2.1 PM Sample Collection and Extraction

PM_2.5_ samples were collected at Davis, CA (38.5449° N, 121.7405° W, ∼15 miles southwest of Sacramento) from November 2019 to October 2020. Particles were collected on precleaned (shaking gently in Milli-Q water for 8 h and dried at 100 °C) Teflon-coated quartz filters using a high-volume sampler equipped with a PM_10_ inlet (Graseby Andersen) and two offset, slotted impactor plates (Tisch Environmental, Inc., 230 series) to remove particles larger than 2.5 μm. The air flow rate was held at 68 (±2) m^3^ h^–1^. Each sample was collected for 24 h or 1 week continuously (Table S1). After sampling, a filter was wrapped in prebaked (500 °C, 8 h) aluminum foil and placed in a desiccator before cold storage. Afterward, the foil-wrapped filter was sealed in a Ziplock bag and stored at −20 °C until extraction. We expect the airtight Ziploc bags can help maintain a relatively low humidity and prevent water condensation within the bag. On the day of filter extraction, the samples were also placed in the desiccator after removal from the freezer to prevent water condensation. The extraction procedure includes cutting a 2 cm × 2 cm square from the filter, placing it in an amber glass vial with 1.0 mL of Milli-Q water, and shaking for 4 h on a shaker (OS-500, VWR) in the dark. Afterward, the water extract was filtered (0.22 μm PTFE), flash frozen using liquid N_2_ and stored at −20 °C until use. The extraction procedure produces dilute extracts that correspond to equivalent liquid water contents (LWC) for the PM in the range of 6.6−65.7 mg-H_2_O m^–3^-air (Table S1), i.e., relatively concentrated cloud and fog drops.

### 2.2 Chemical and Optical Analyses of PM Extracts

PM extracts were analyzed for (1) mass concentrations and mass spectra of water-soluble organics, sulfate, nitrate, ammonium, and chloride using a high-resolution time-of-flight aerosol mass spectrometer (AMS, Aerodyne Res. Inc.); (2) light absorbance (200–800 nm) using a UV–vis spectrophotometer (UV-2501PC, Shimadzu); (3) major anions (F^–^, Cl^–^, Br^–^, NO_3_^–^, PO_4_^3–^, SO_4_^2–^, and formate) and cations (Li^+^, Na^+^, NH_4_^+^, K^+^, Ca^2+^, and Mg^2+^) using two ion chromatographs equipped with conductivity detectors (881 Compact IC Pro, Metrohm); and (4) water-soluble organic carbon (WSOC) using a total organic carbon analyzer (TOC-VPCH, Shimadzu). Prior to AMS analysis, the PM extracts were spiked with isotopic ^34^sulfate (^34^SO_4_^2–^) as an internal standard and nebulized in argon (Ar, industrial grade, 99.997%) using a micronebulization assembly.^49^ The AMS was operated in the “V” mode (mass resolutions of ∼3000) to acquire mass spectra up to *m*/*z* = 425 amu. AMS analyzes nonrefractory aerosol species that evaporate at ∼600 °C under high vacuum via 70 eV EI mass spectrometry.^50,51^

### 2.3 Measurements of Photooxidants

The concentrations of three photooxidants (·OH, ^1^O_2_*, and ^3^C*) were measured in the illuminated PM extracts as described in Ma et al. (2023).^47^ Briefly, the PM extract was spiked with an oxidant probe, transferred into a capped quartz tube (5 mm inner diameter). The sample was subjected to illumination at 20 °C with a 1000 W xenon arc lamp fitted with a water filter to reduce sample heating, an AM1.0 air mass filter, and a 295 nm long-pass filter to simulate tropospheric sunlight. At regular intervals, small aliquots of the illuminated sample (and the corresponding dark control) were collected and analyzed by a high-performance liquid chromatograph (HPLC) equipped with a UV–vis detector to determine the probe concentration. Benzoic acid (BA) was used as the probe to quantify ·OH via BA loss and para-hydroxybenzoic acid formation. The ^1^O_2_* concentration was quantified using furfuryl alcohol (FFA) as the probe and deuterium oxide (D_2_O) as a diagnostic tool. The ^3^C* concentration was measured using syringol (SYR) as the probe and accounted for probe inhibition. Oxidant concentrations are normalized to midday sunlight at Davis on the winter solstice, i.e., solar zenith angle = 62°.^47^

### 2.4 Data Analysis

#### 2.4.1 UV–Vis Absorption Properties

The light absorption coefficient (α_λ_, cm^–1^) of each PM extract was calculated as
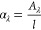
1where *A*_λ_ is the measured base-10 light absorbance of the PM extract at wavelength λ, and *l* is the path length of the cuvette (1 cm). The mass absorption coefficient (MAC_λ_, m^2^ g^–1^) of the PM extract was calculated as
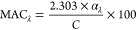
2where *C* is the WSOA mass concentration (mg L^–1^) in the PM extract measured by AMS, 2.303 is the factor to convert from log_10_ to natural log, and 100 is for unit conversion. The rate of sunlight absorption of the PM extract (*R*_abs_, mol photons L ^–1^ s ^–1^) in the range of 290–500 nm was calculated as
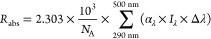
3where *I*_λ_ is the midday Davis winter-solstice actinic flux (photons cm^–2^ s^–1^ nm^–1^) from the Tropospheric Ultraviolet and Visible (TUV) Radiation Model version 5.3 (https://www.acom.ucar.edu/Models/TUV/Interactive_TUV/), Δλ is the interval between adjacent wavelengths in the TUV output, 2.303 is for base conversion between log_10_ and natural log, 10^3^ is for unit conversion, and *N*_A_ is Avogadro’s number. The absorption Ångström exponent (AAE) of the PM extract was determined in the wavelength of 290–500 nm by eq 4:
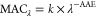
4where *k* is a wavelength-independent constant.

#### 2.4.2 AMS Data Treatment and Quantification of PM Species

The AMS data were processed using the standard analysis toolkits (SQUIRREL v1.65C and PIKA 1.25C). The organic water signals were parametrized using the standard method for HR-AMS ambient data processing: H_2_O^+^ = 0.225 × CO_2_^+^, HO^+^ = 0.25 × H_2_O^+^, and O^+^ = 0.04 × H_2_O^+^.^52,53^ Due to the use of Ar, the CO^+^ signal was quantified directly in the AMS.^19^ The atomic ratios of oxygen-to-carbon (O/C), hydrogen-to-carbon (H/C) and organic mass-to-carbon ratio (OM/OC) in the WSOA were calculated using the Aiken-Ambient method.^52^

By using the ^34^SO_4_^2–^ internal standard, concentrations of water-soluble PM components (i.e., sulfate, nitrate, organics, ammonium, and chloride) can be quantitatively determined via AMS analysis.^49^ The concentration of species X in PM extract solution ([X]_solution_, μg mL^–1^) was calculated as
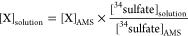
5where [X]_AMS_ and [^34^sulfate]_AMS_ are the AMS-measured concentrations (μg m^–3^) of X and the spiked ^34^SO_4_^2–^, respectively, in the aerosolized PM extract, and [^34^sulfate]_solution_ is the known concentration (μg mL^–1^) of the ^34^SO_4_^2–^ internal standard in the PM extract.

Next, the ambient concentration of X ([X]_ambient_, μg m^–3^) was calculated as
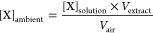
6where *V*_extract_ is the volume (mL) of the PM extract solution, and *V*_air_ is the volume (m^3^) of air sampled by a square cut of filter.

Figure S1 shows the comparisons of the AMS measured concentrations of water-soluble species versus those by IC measurements. AMS and IC agreed reasonably well for the measurements of sulfate concentration, while the nitrate concentration was ∼2 times lower in AMS, likely due to evaporation of NH_4_NO_3_ during aerosol generation prior to AMS measurement. Thus, the IC-measured nitrate concentrations are reported in this study. In addition, as shown in Figure S2, the AMS measured organic carbon concentrations agree well with the TOC measurements.

#### 2.4.3 PMF Analysis of Combined AMS Mass Spectra and UV–Vis Absorption Spectra

To understand the chemical composition and light absorption properties of WSOA components from different sources, positive matrix factorization (PMF) was performed on the combined matrix of the AMS spectra and the UV–vis spectra of the PM extracts. The AMS spectral matrix includes the high resolution mass spectra (HRMS) of organic ions between *m*/*z* 12–120, selected phenolic tracer ions with *m*/*z* > 120, including C_7_H_5_O_2_^+^, C_7_H_8_O_3_^+^, C_8_H_7_O_3_^+^, C_7_H_8_O_4_^+^, C_8_H_10_O_4_^+^, C_10_H_12_O_4_^+^, C_14_H_11_O_3_^+^, C_14_H_14_O_4_^+^, C_14_H_14_O_5_^+^, C_18_H_14_O_4_^+^, C_18_H_17_O_5_^+^, C_16_H_18_O_7_^+^, C_20_H_22_O_6_^+^, C_21_H_20_O_7_^+^,^19,39^ and major inorganic ions, including SO_*x*_^+^ ions (i.e., SO^+^, SO_2_^+^, HSO_2_^+^, SO_3_^+^, HSO_3_^+^, H_2_SO_4_^+^) and NO_*x*_^+^ ions (i.e., NO^+^ and NO_2_^+^),^54^ and the unit mass solution (UMR) spectral signals at *m*/*z* 121–425. The UV–vis spectral matrix includes the absorption spectra in the range of 290–500 nm. To account for the proportional relationship between the phenolic tracer ions with *m*/*z* > 120 and their corresponding UMR signals, we applied downweighing to these phenolic ions by multiplying their error values by a factor of sqrt (2). The PMF results were evaluated using the PMF Evaluation Toolkit (PET v3.08 downloaded from http://cires1.colorado.edu/jimenez-group/wiki/index.php/PMF-AMS_Analysis_Guide). The 5-factor solution with fPeak = 0 was chosen based on the evaluation criteria.^55,56^ A summary of the diagnostic plots for the 5-factor PMF solution is presented in Figure S3. The calculations of organic and inorganic species concentrations and mass absorption coefficients for the PMF factors are presented in Section S1.

#### 2.4.4 Estimation of the Oxidant Formation Potentials of the WSOA Factors

To estimate the oxidant formation potentials of different WSOA factors, multilinear regression was performed to model the relationship between oxidant concentrations measured in the illuminated dilute PM extracts (i.e., [·OH], [^1^O_2_*], or [^3^C*]; mol/L) and the concentrations of the five PMF factors (i.e., [_WS_BBOA_fresh_], [_WS_BBOA_aged_], [_WS_OOA_1_], [_WS_OOA_2_], and [_WS_OOA_3_]; mg/L) by fitting the following linear equation:
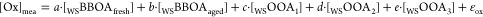
7where [Ox]_mea_ is an array of the concentration of a given oxidant measured in the illuminated PM extracts, *a*–*e* are the least-squares fitting parameters, and ε_ox_ is the residual vector. Here, *a*–*e* are in units of mol-oxidant/mg-organic, representing the oxidant production potentials (PP_Ox_) of the corresponding WSOA factors. ε_ox_ (mol/L) represents the differences between the measured and the modeled oxidant concentrations. Since oxidant concentrations vary nonlinearly with extract dilution,^12,47^ our PP_Ox_ values can only be applied to conditions similar to the relatively dilute extract conditions of our measurements; i.e., our oxidant predictions here apply to fog/cloud conditions but not to the more concentrated case of aerosol liquid water.

## 3 Results and Discussion

### 3.1 Bulk Composition and Light Absorption Properties of WSOA in PM_2.5_

**Figure 1:**
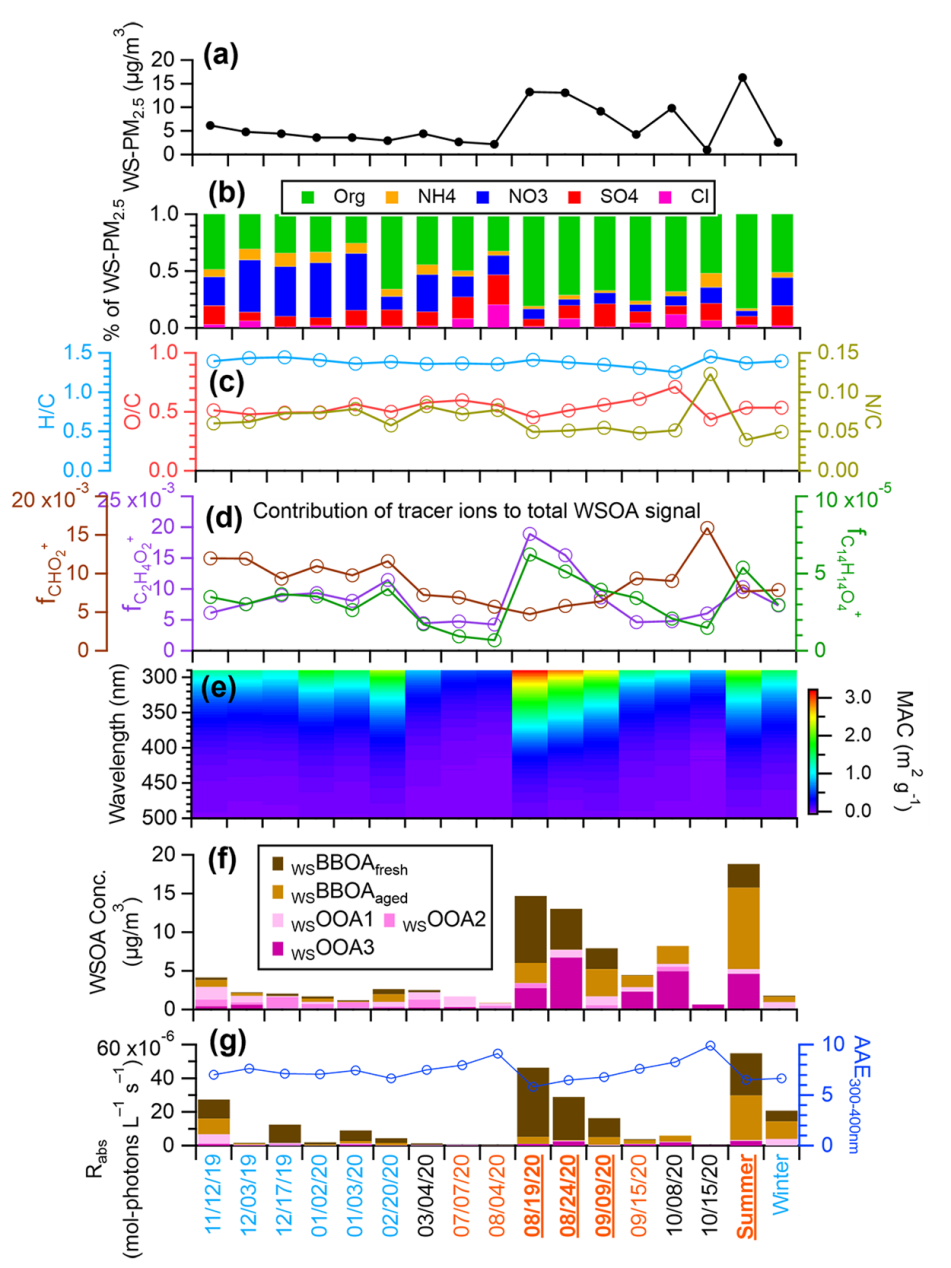
Characteristics of the 17 samples we studied: (a) Ambient mass concentration of water-soluble PM_2.5_ (WS-PM_2.5_). (b) Composition of WS-PM_2.5_. (c) Elemental ratios of the water-soluble organic aerosol (WSOA). (d) Contribution of selected AMS tracer ions to the total WSOA signal. (e) Mass absorption coefficient (MAC) of the WSOA. (f) Ambient concentrations of the five WSOA factors resolved from positive matrix factorization (PMF). (g) Rate of sunlight absorption (*R*_abs_) contributed by each WSOA factor and AAE of the WSOA. The *x*-axis shows the PM sampling dates and the colors denote the seasons: winter (blue), summer (orange), and spring and fall (black). Samples significantly influenced by wildfire plumes are underlined. The last two samples are a composite summer sample and a composite winter sample, respectively. Details about the PM_2.5_ samples are in Table S1.

A total of 17 Davis PM_2.5_ aqueous extracts were characterized, of which 7 were collected during summer, 7 during winter, one during spring and two during fall (Table S1). To confirm the collected PM_2.5_ samples are representative of all the days from November 2019 to October 2020, Kolmogorov–Smirnov test was performed on the daily ambient PM_2.5_ data (measured at UC Davis sampling site by California Air Resources) using the built-in function in IGOR Pro 8 (WaveMetrics) (Figure S5). A majority of the samples were influenced by biomass burning, including four of the summer PM extracts that were significantly impacted by wildfire smoke and all of the winter samples which were affected by residential wood burning. Figure 1 summarizes the chemical composition and light absorption properties of the water-soluble PM_2.5_ (WS-PM_2.5_) components. The WS-PM_2.5_ concentration is in the range of 1.0–16.3 μg m^–3^ (Figure 1a). Organics are a dominant component, accounting for 26–83% of the WS-PM_2.5_ mass, while nitrate contributes substantially (up to 50%) during winter (Figure 1b). The WSOA is moderately oxidized, with O/C ratios in the range of 0.43–0.71 and H/C ratios in the range of 1.25–1.45 (Figures 1c and S6c). Figure 1d shows the mass fractions of three AMS tracer ions: CHO_2_^+^ (*m*/*z* = 44.998; a marker for carboxylic acids^39^), C_2_H_4_O_2_^+^ (*m*/*z* = 60.021; a tracer for anhydrous sugars such as levoglucosan^57^), and C_14_H_14_O_4_^+^ (*m*/*z* = 246.089; a tracer for phenolic aqueous secondary organic aerosol (aqSOA)^19^). The fractional contribution of CHO_2_^+^ to the total WSOA signal (f_CHO2+_) ranges between 0.5–1.5% in the samples, suggesting a relatively constant content of carboxylic acids in the WSOA. However, both f_C2H4O2+_ and f_C14H14O4+_ are significantly elevated in the wildfire-influenced samples and in the winter samples (Figures 1d and S6b), indicating contributions of primary and secondary BBOAs. In addition, as shown in Figures 1e and 1g, the wildfire-influenced samples are much more light-absorbing, showing higher MAC_365 nm_ (up to 1.1 m^2^ g^–1^) and lower AAE (down to 5.8), than the other samples whose MAC_365 nm_ are in the range of 0.08–0.55 and the AAE are in the range of 6.8–9.9. These results are consistent with previous findings that biomass burning is an important source of BrC in the atmosphere.^3^

### 3.2 Chemical Compositions and Light Absorption of WSOA Factors

**Figure 2:**
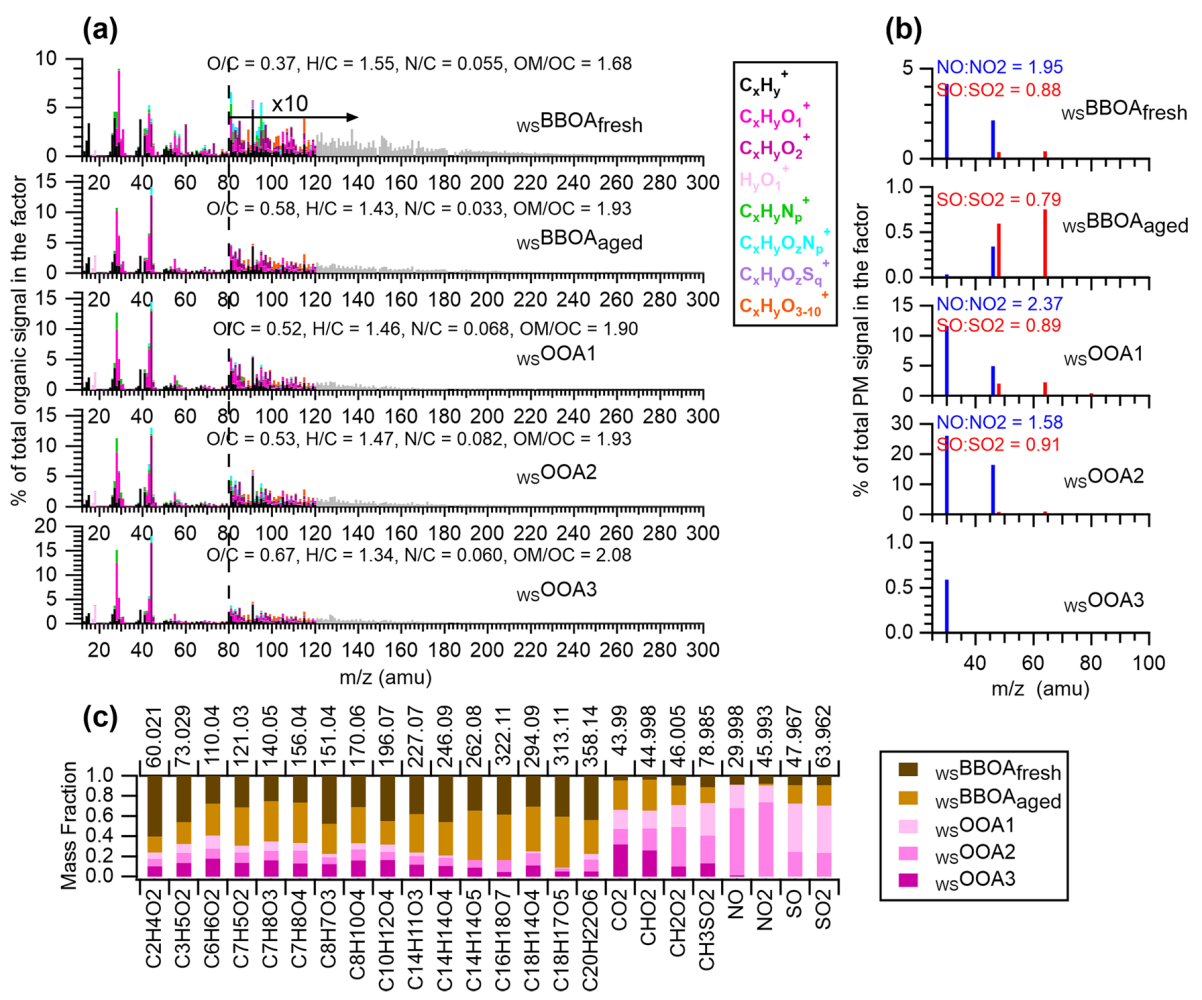
(a) Mass spectra of the five WSOA factors colored by ion families. HRMS ions are included for *m*/*z* < 120, and UMR signals (in gray) for *m*/*z* > 120. (b) Signals of NO^+^, NO_2_^+^, SO^+^, and SO_2_^+^ ions in the PMF-resolved WS-PM_2.5_ factors. (c) Mass fraction of selected AMS tracer ions attributed to each WSOA factor.

Performing PMF analysis on the combined AMS and UV–vis absorption data of the PM extracts (see Section 2.4.3 for more details) resolved five distinct WSOA factors. The first two factors are closely related to biomass burning and are denoted as fresh water-soluble BBOA (_WS_BBOA_fresh_; O/C = 0.37, MAC_365 nm_ = 1.1 m^2^ g^–1^, AAE = 5.5) and more aged _WS_BBOA_aged_ (O/C = 0.58, MAC_365 nm_ = 0.25 m^2^ g^–1^, AAE = 7.1). The other three factors are called water-soluble oxygenated OA (_WS_OOA), specifically, _WS_OOA1 (O/C = 0.52, MAC_365 nm_ = 0.10 m^2^ g^–1^, AAE = 6.9), _WS_OOA2 (O/C = 0.53, MAC_365 nm_ = 0.01 m^2^ g^–1^, AAE = 11.1), and _WS_OOA3 (O/C = 0.67, MAC_365 nm_ = 0.10 m^2^ g^–1^, AAE = 8.8). The differentiation between _WS_BBOAs and _WS_OOAs is mainly made based on characteristic mass spectral features: both _WS_BBOAs show enhanced ion signals indicative of biomass burning influence, such as C_2_H_4_O_2_^+^ and C_3_H_5_O_2_^+^ (tracer ions for levoglucosan) and C_6_H_6_O_2_^+^, C_8_H_10_O_4_^+^, C_14_H_14_O_4_^+^ (tracer ions for phenols) (Figure 2a), whereas the mass spectra of _WS_OOAs demonstrate more prominent oxygenated ions (e.g., CO_2_^+^ and CHO_2_^+^) (Figure 2c). In addition, both _WS_OOA1 and _WS_OOA2 are associated with substantial amounts of secondary inorganic species, while _WS_OOA3 is not (Figure 2b). _WS_OOA3 appears closely linked to BB emissions, even though it does not contain levoglucosan-related tracers: _WS_OOA3 concentrations are considerably elevated in wildfire-influenced samples (Figure 1f) and contains ions representing oxidation products of phenols. More details are discussed below.

**Figure 3:**
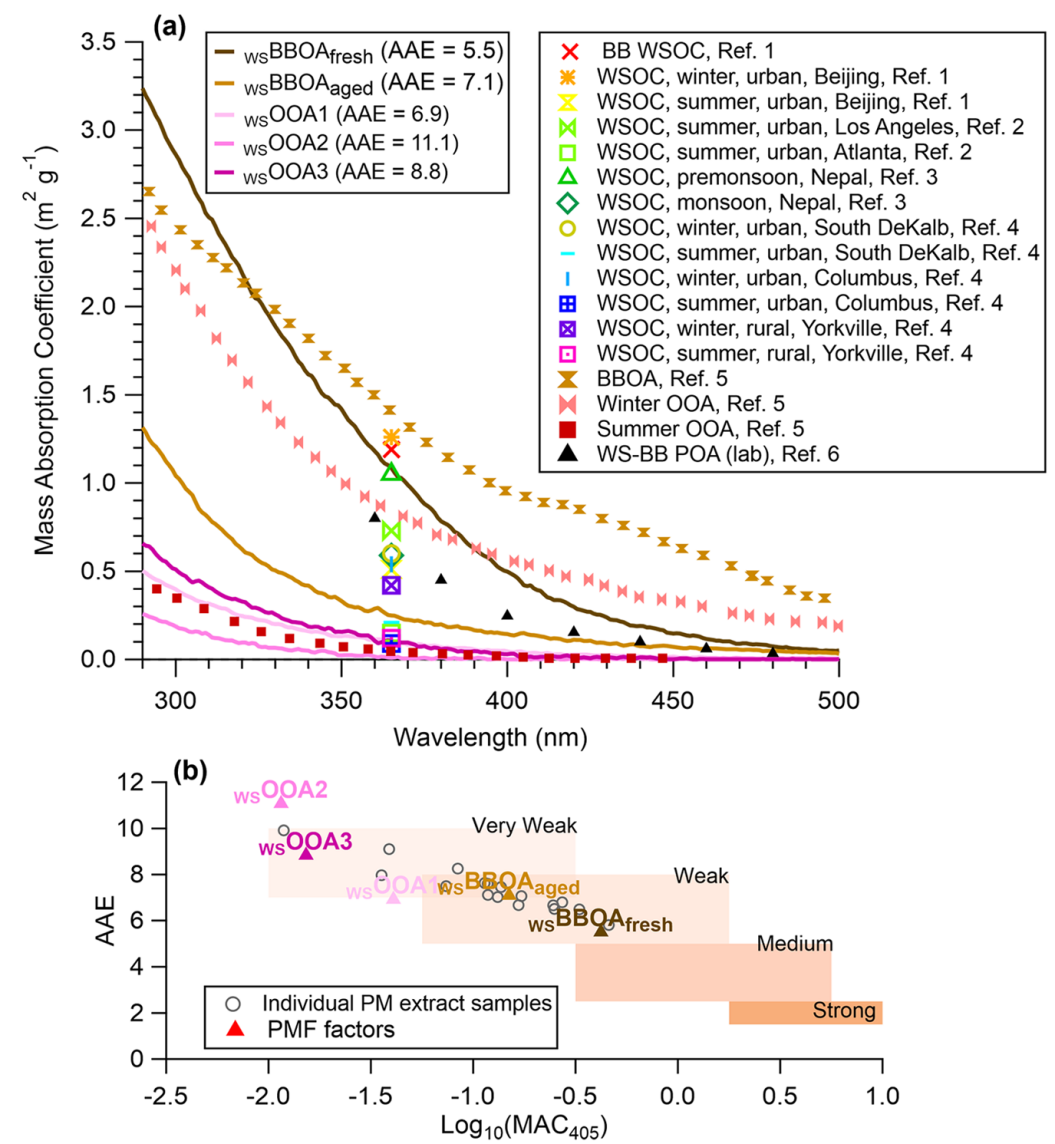
(a) Comparisons of the mass absorption coefficients (MAC) of the five WSOA factors resolved in this study with previously reported values. Ref. 1: Du et al. (2014) (ref (60)); Ref. 2: Zhang et al. (2011) (ref (61)); Ref. 3: Wu et al. (2019) (ref (62)); Ref. 4: Hecobian et al. (2010) (ref (4)); Ref. 5: Moschos et al. (2018) (ref (40)); Ref. 6: Chen and Bond (2010) (ref (63)). (b) Optical-based classification of different BrC components in the AAE vs log_10_(MAC_405 nm_) space.^64−66^ The shaded regions represent “optical bins” for very weakly absorbing, weakly absorbing, moderately absorbing, and strongly absorbing BrC. The gray circles represent individual PM extracts, and the solid triangles represent the water-soluble OA factors obtained from the PMF analysis in this study.

Figures 2a,b present the AMS mass spectra of the WSOA factors and Figure 3a presents their mass absorption coefficient spectra. Among the five WSOA factors, _WS_BBOA_fresh_ is the least oxidized and demonstrates mass spectral features of fresh BBOA that have been observed in the field.^58,59^ In the _WS_BBOA_fresh_ mass spectrum, f_C2H4O2+_ (2.9%), f_C3H5O2+_ (1.1%), and high mass ions (e.g., f_*m*/*z*>120_ = 10%; Figure 2a) are significantly enhanced, indicating enrichments of anhydrous sugars and high molecular weight species. In addition, _WS_BBOA_fresh_ is associated with a moderately enhanced nitrate signal with an NO^+^/NO_2_^+^ ratio of 1.95 (Figure 2b). This ratio is close to the NO^+^/NO_2_^+^ in pure ammonium nitrate (1.81), suggesting that _WS_BBOA_fresh_ is mainly associated with inorganic nitrate. This finding is consistent with the rapid conversion of NO_*x*_ to nitrate in fresh BB smoke.^58^ Furthermore, _WS_BBOA_fresh_ is elevated in the wildfire-influenced summer samples (as identified by airmass back trajectories, Figure S7) and in the winter samples, a period when residential wood burning is common in Davis (Figure 1f).

**Figure 4:**
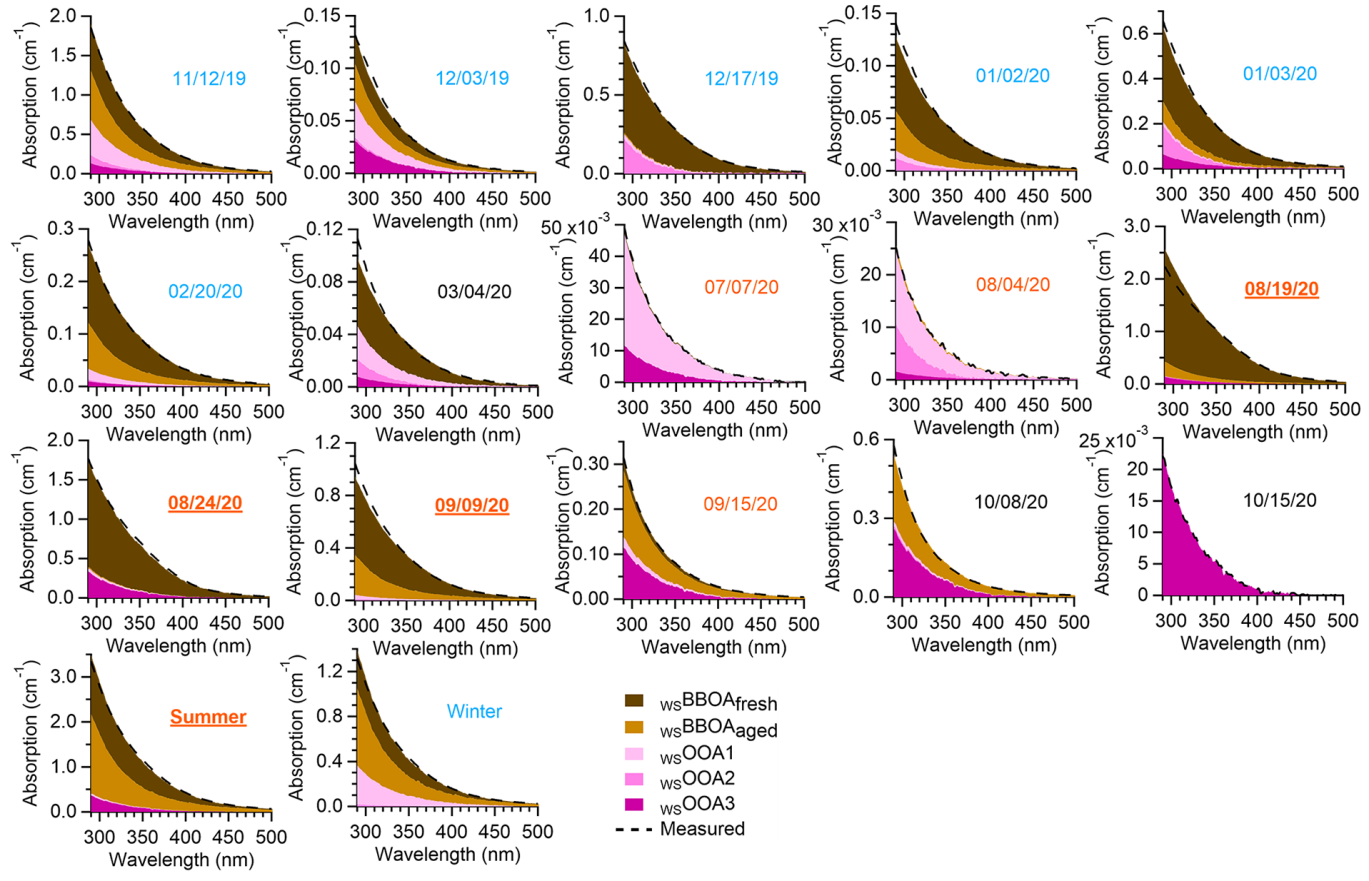
Contributions of the five WSOA factors to the total light absorption in each PM_2.5_ extract. Details about the samples are in Table S1.

_WS_BBOA_fresh_ is the most light-absorbing factor, with the highest MAC and the lowest AAE among the five WSOA factors (Figures 3). BBOA contains light-absorbing compounds such as nitro-organics (RNO_2_), organonitrates (RONO_2_), polycyclic aromatic hydrocarbon (PAH) derivatives, and polyphenols.^67,68^ As summarized in Figure 3a, the MAC values of _WS_BBOA_fresh_ are comparable to those measured in ambient WSOA influenced by BB^60^ and in lab-generated water-soluble primary BBOA.^63^ Moschos et al. performed PMF on combined AMS and UV–vis data for water-soluble PM from Switzerland and retrieved three factors: BBOA, winter OOA, and summer OOA. They also found that their _WS_BBOA factor is substantially more absorbing than the _WS_OOAs.^40^ The absorptivity of the Moschos _WS_BBOA is significantly higher than our _WS_BBOA_fresh_ in the visible light range but the two are comparable in the UV region (Figure 3a). A possible reason for this discrepancy is that our _WS_BBOA_fresh_ was more strongly influenced by summer-time wildfires while the Moschos _WS_BBOA mainly represented wintertime residential wood combustion. Indeed, the light absorptivity of BBOA can be influenced by factors such as biomass types, burning conditions, and the aging of BBOA.^63,69,70^_WS_BBOA_fresh_ accounts for a significant fraction of the total light absorption in Davis, contributing an average of 61% of the total rate of sunlight absorption in the wavelength lower than 500 nm in all the PM_2.5_ extracts and as high as 89% in wildfire-influenced samples (Figures 4 and 1g). Similar findings were reported previously in the southeastern U.S., where biomass burning dominates BrC absorption at both rural and urban sites.^4,71^

The mass spectral profile of _WS_BBOA_aged_ is similar to those of aged BBOA factors observed in previous studies.^58,59,72^ As shown in Figures 2a and 3, the aged BB factor is more oxidized and less absorbing than _WS_BBOA_fresh_ and contains a lower level of anhydrous sugars (f_C2H4O2+_ = 0.64% vs 2.9% in _WS_BBOA_fresh_) but a higher content of carboxylates (f_CHO2+_ = 0.92% vs 0.13% in _WS_BBOA_fresh_). In addition, _WS_BBOA_aged_ correlates well with phenolic SOA tracer ions such as C_6_H_6_O_2_^+^, C_7_H_5_O_2_^+^, C_7_H_8_O_3_^+^, C_7_H_8_O_4_^+^, C_8_H_7_O_3_^+^, C_14_H_14_O_4_^+^, C_14_H_14_O_5_^+^, C_16_H_18_O_7_^+^, C_18_H_17_O_5_^+^, and C_20_H_22_O_6_^+^ (Figures S10 and 2c). These results suggest that _WS_BBOA_aged_ represents more aged BB smoke and contains oxidation products from BB-emitted phenols.^19,39,73−75^ The MAC of _WS_BBOA_aged_ is comparable to that of wintertime WSOA measured at a rural site in the southeast US, where BB was identified as a major source of BrC.^4^ On average, _WS_BBOA_aged_ accounts for 28% of the rate of sunlight absorption of the PM extracts (Figure 1g). The lower light absorptivity of _WS_BBOA_aged_ compared to _WS_BBOA_fresh_ might indicate photobleaching during the aging of BBOA.^76^

The _WS_OOAs are less light absorbing than the _WS_BBOAs (Figures 3) and have negligible contributions from primary BBOA, as indicated by low f_C2H4O2+_ in their mass spectra (Figure 2c). But they show enhanced f_CHO2+_ (Figures 2a and 2c), suggesting that _WS_OOAs are relatively more enriched in organic acids. The mass fractions of _WS_OOA1 and _WS_OOA2 in PM are greater during wintertime, while the more oxidized _WS_OOA3 is more abundant during summertime (Figure 1). A majority of the nitrate and sulfate in PM is associated with _WS_OOA1 and _WS_OOA2, but in different mass ratios. Close to 50% of the total sulfate mass is associated with _WS_OOA1 while nearly 80% of the nitrate mass is associated with _WS_OOA2 (Figure 2c). In addition, CH_3_SO_2_^+^ (a tracer ion for methanesulfonic acid (MSA)^77^) is enriched in _WS_OOA1. Although MSA is commonly associated with oceanic sources, previous studies have found MSA in boundary layer OOA^58^ and it can be an aqueous-phase SOA product of S-containing VOCs from terrestrial sources.^59,77,78^ These results suggest that _WS_OOA1 likely represents aqSOA. _WS_OOA1 is more light-absorbing than _WS_OOA2 and demonstrates MAC values similar to summertime ambient WSOA observed in rural Yorkville and in urban Columbus,^4^ as well as the Summer OOA resolved from PMF analysis of water-soluble PM from Switzerland.^40^ Compared to _WS_OOA1 and _WS_OOA2, _WS_OOA3 is more oxidized (O/C = 0.67) and more light-absorbing (Figures 2 and 3). The association of _WS_OOA3 with a small signal of NO^+^ but almost no NO_2_^+^ (Figure 2c) suggests that _WS_OOA3 may contain a small amount of organonitrate and nitro compounds, which could be produced in aged BB plumes or from gas phase photooxidation of urban emissions under high NO_*x*_ conditions.^29,79^ In addition, the mass spectrum of _WS_OOA3 shows a resemblance to the spectra of secondary BBOAs observed in aged wildfire smokes.^58,72^ These observations, together with the increase of this factor in the BB-influenced samples, suggest that _WS_OOA3 is linked to SOA of BB origins. The _WS_OOAs together account for 11% of the total sunlight absorption of the PM extracts in Davis (Figures 4 and 1g).

### 3.3 Relationship between WSOA Components and Condensed-Phase Oxidants (·OH, ^3^C*, and ^1^O_2_*)

**Figure 5:**
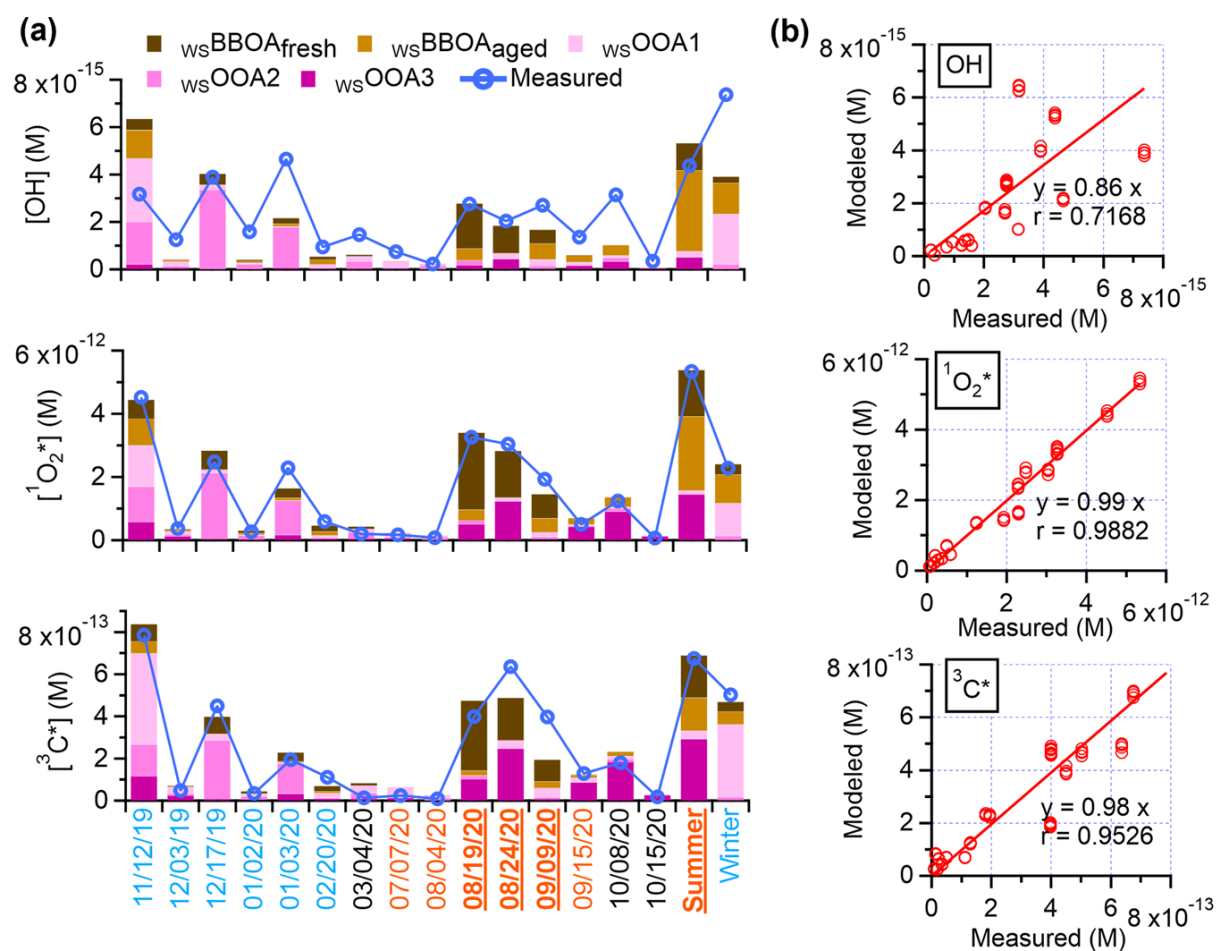
(a) Estimated concentrations of oxidants (·OH, ^1^O_2_*, and ^3^C*) contributed by each WSOA factor, along with measured oxidant concentrations in illuminated PM_2.5_ extracts. (b) Scatter plots compare the modeled oxidant concentrations versus measured values. Details of each PM_2.5_ sample are in Table S1.

Figure 5a displays the steady-state concentrations of ·OH, ^1^O_2_* and ^3^C* in the PM extracts illuminated under simulated sunlight. The average concentrations of ·OH, ^1^O_2_* and ^3^C* in the PM extracts are 2 × 10^–15^ M, 2 × 10^–12^ M, and 3 × 10^–13^ M, respectively. These values are comparable with previously reported ^1^O_2_* and ^3^C* concentrations in dilute particle extracts collected in Davis, but are about 5 times higher for ·OH.^12^ Since WSOA is both a potential source and an important sink of the oxidants,^80,81^ the aqueous-phase oxidant concentrations can be highly dependent on both the composition and the extent of dilution of the WSOA.^12,47,48^

To explore the dependencies of ^3^C*, ^1^O_2_*, and ·OH formation on WSOA composition, we performed multilinear regression analysis to model the measured oxidant concentrations as a linear combination of the concentrations of the five WSOA factors (see Section 2.4.4 for details). Figure 5a shows the modeled concentrations of ·OH, ^1^O_2_*, and ^3^C* according to the contributions of the five WSOA factors in each PM extract, and Figure 5b shows the correlations between the modeled and the measured oxidant values. While ^1^O_2_* and ^3^C* can be properly modeled by the WSOA factors, the correlation between modeled and measured ·OH is relatively poor (Pearson’s *r* = 0.72; Figure 5b). These results suggest that photoreactions of BrC components in WSOA are important sources of ^1^O_2_* and ^3^C*, whereas ·OH may have other major sources, such as nitrite and nitrate photolysis,^15,82^ photo-Fenton reactions,^83^ and peroxides.^84−86^ The lack of correlation between measured and modeled ·OH may also indicate the more intricate sinks/consumption pathways of ·OH. In the aqueous phase, a large variety of organic compounds can react rapidly with ·OH at nearly diffusion-controlled rates and serve as important sinks for ·OH, including alcohols, halogenated alkanes, amines, aromatic compounds, and inorganic species.^81^

**Figure 6:**
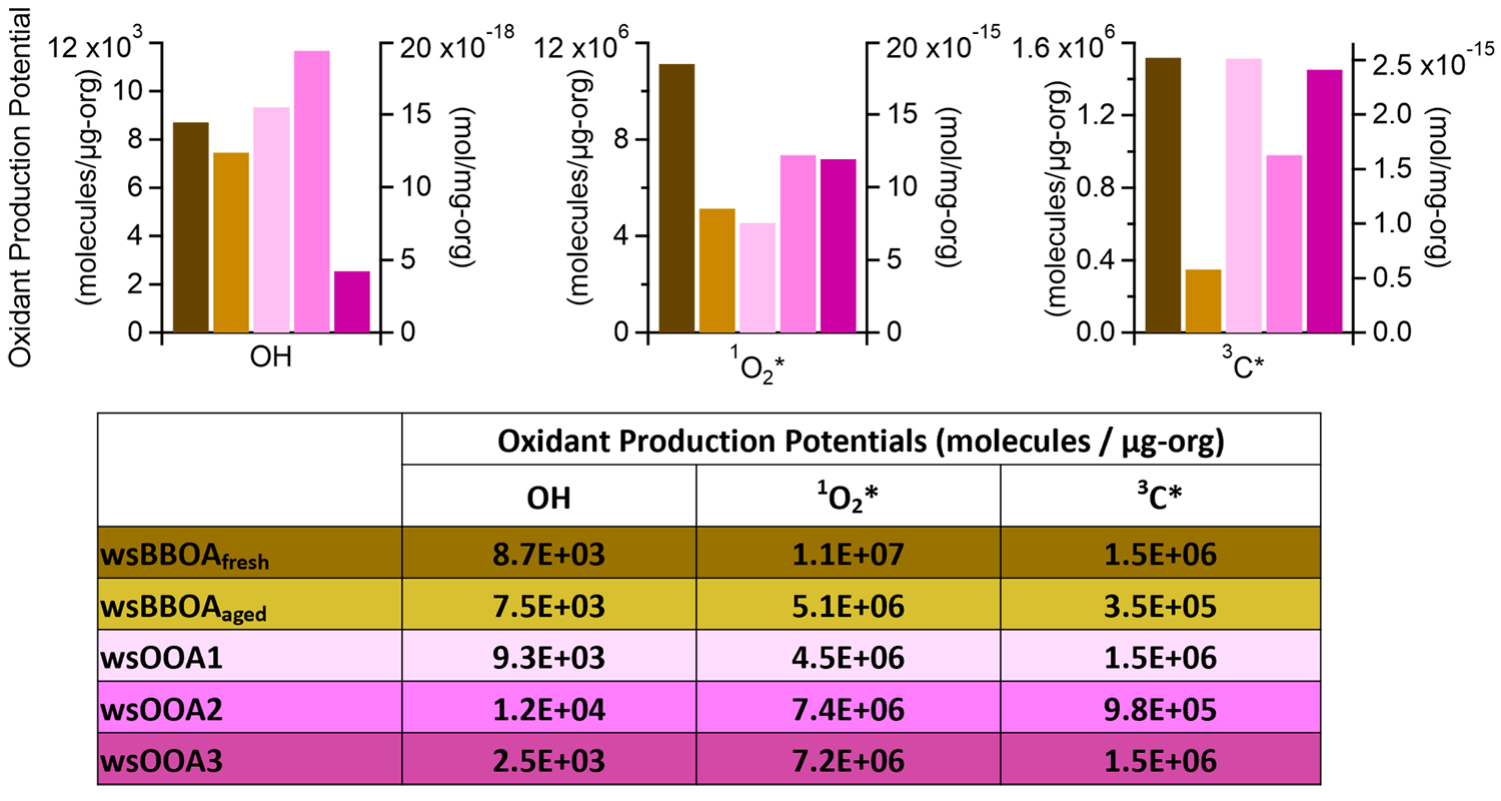
Estimated photoproduction potentials of ·OH, ^1^O_2_*, and ^3^C* by each WSOA factor under PM extract conditions.

The least-squares fitting parameters derived from the multilinear analysis have units of mol-oxidant/mg-organic and thus represent the oxidant production potentials of individual WSOA factors under cloud/fog conditions. As shown in Figure 6, among the five WSOA factors, _WS_BBOA_fresh_ demonstrates the highest ^1^O_2_* and ^3^C* production potentials: 1.9 × 10^–14^ and 2.5 × 10^–15^ mol/mg-organic, or 1.1 × 10^7^ and 1.5 × 10^6^ molecules/μg-organic, respectively. This result is consistent with _WS_BBOA_fresh_ being the most light-absorbing factor and suggests this factor contains abundant BrC precursors for ^1^O_2_* and ^3^C*. These chromophore precursors likely include BB-emitted aromatic carbonyls, which absorb sunlight to produce ^3^C*^12,16,87,88^ that in turn can transfer energy to ground state dissolved O_2_ to form ^1^O_2_*.^89^ However, although the MAC_365 nm_ of _WS_BBOA_fresh_ is 4–100 times higher than the other WSOA factors, its potential to produce ^1^O_2_* and ^3^C* is only 1–4 times higher. This suggests that the BrC chromophores in _WS_BBOA_fresh_ are less efficient sources of ^1^O_2_* and ^3^C*, i.e., have lower quantum yields.^48^ The ^3^C* production potentials of _WS_OOA1 and _WS_OOA3 are comparable to that of _WS_BBOA_fresh_, suggesting that oxygenated organic species can also be potent sources of ^3^C*. This observation is consistent with the fact that _WS_OOAs also contain BrC components. Figure S11 shows the modeled fractional contribution of individual WSOA factors to the total oxidant concentrations in the PM extracts. On average, the two _WS_BBOA factors together account for 46%, 50%, and 34% of the ·OH, ^1^O_2_*, and ^3^C* concentrations in the illuminated PM extracts whereas the three _WS_OOA factors together account for 54%, 50%, and 66%. This result suggests that both BB smoke and oxygenated organic species, some of which are derived from BB species, are important sources of aqueous-phase oxidants in northern California.

Figure S12 shows the correlation coefficients between oxidant concentrations and AMS-measured WSOA ion families. While ^1^O_2_* and ^3^C* correlate well with all of the ion families (*r* = 0.91–0.98), ·OH shows lower correlations (*r* = 0.64–0.70). This result supports the idea that ^1^O_2_* and ^3^C* are mainly formed from photoexcitation of chromophoric organics while ·OH has more varied photochemical sources in the aqueous phase. In addition, both ^1^O_2_* and ^3^C* show high correlations with N-containing ions, consistent with previous findings that N-containing compounds, such as nitrophenols^93−95^ and imidazoles^78,96^ represent an important class of BrC species.

### 3.4 Estimation of Global Aqueous-Phase Oxidant Concentrations and Atmospheric Implications

Based on our extraction conditions, the equivalent LWC values of the PM extracts were in the range of 6.6–65.7 mg m^–3^, i.e., concentrated cloud and fogwater conditions. Thus, using the oxidant production potentials (PP_Ox_) derived for the WSOA factors (Figure 6), we can estimate the concentrations of ·OH, ^1^O_2_*, and ^3^C* in cloud/fog waters based on BBOA and OOA concentrations in previous studies:^55,58,59,72,97−99^
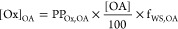
8

In this equation, [Ox]_OA_ (mol m^–3^) is the estimated aqueous-phase oxidant concentration contributed by an WSOA factor, PP_Ox,OA_ (mol-oxidant/mg-organic) is the oxidant production potential of a WSOA factor derived through eq 7, [OA] (μg m^–3^) is the ambient OA concentration, and f_ws,OA_ is the mass fraction of water-soluble components in an OA factor. Values of f_ws,OA_ for BBOA and OOA were estimated as 37% and 49%, respectively.^100^ We used the PP_Ox_ of _WS_BBOA_fresh_ to represent fresh _WS_BBOA, the PP_Ox_ of _WS_BBOA_aged_ to represent oxidized _WS_BBOA, and the average value of _WS_OOA1, _WS_OOA2, and _WS_OOA3 to represent _WS_OOA in the ambient locations. Note that modeled PP_Ox_ of _WS_BBOAs and _WS_OOAs in this study only represent values under dilute conditions (e.g., cloud and fog) and are generally not applicable to aerosol liquid water (ALW) conditions. This is because of two main reasons: (1) the production rates of the oxidants do not always increase linearly as the aqueous phase becomes more concentrated, and (2) the major sinks of the oxidants can differ between dilute and concentrated conditions.^47^ Since the ratio of the production rate to the sink rate constant determines the steady-state concentration of an oxidant, values of PP_Ox_ will vary with dilution factor as PM extracts become more concentrated. In addition, our PP_Ox_ values and oxidant concentrations are for sunlight conditions at midday in Davis on the winter solstice and will vary with location and day/time; for example, Davis photolysis rate constants at midday on the summer solstice are approximately twice as high.

**Figure 7:**
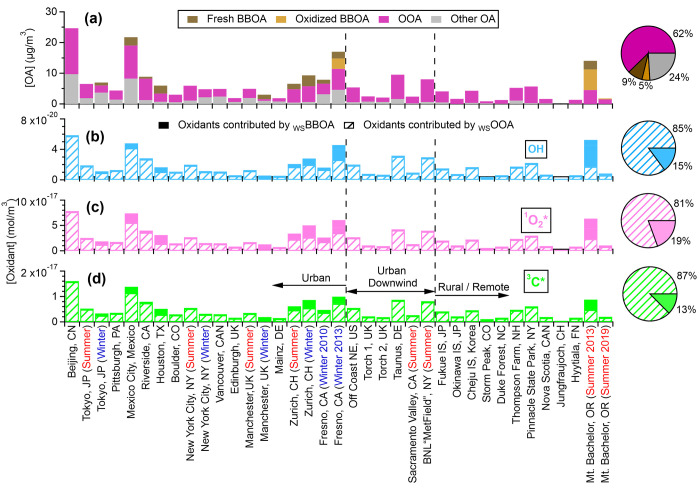
(a) Average concentrations of different OA factors at locations in northern hemisphere and (b–d) estimated cloud/fog concentrations of ·OH, ^1^O_2_*, and ^3^C* contributed by water-soluble BBOA and OOA under winter solstice sunlight. Data in (a) were obtained from previous field observations.^55,58,59,72,97−99^

Figures 7b–d present the estimated ·OH, ^1^O_2_*, and ^3^C* concentrations in atmospheric waters contributed by _WS_BBOA and _WS_OOA at over 30 locations in northern hemisphere. The average estimated ·OH, ^1^O_2_*, and ^3^C* concentrations in the aqueous phase under cloudy/foggy conditions at these locations are 1.5 × 10^–20^ mol m^–3^, 2.2 × 10^–17^ mol m^–3^, and 4.2 × 10^–18^ mol m^–3^, respectively, which are equivalent to 1.5 × 10^–16^ M, 2.2 × 10^–13^ M, and 4.2 × 10^–14^ M, respectively, assuming liquid water content of 0.1 g m^–3^. Although _WS_BBOA shows high PP_Ox_ (especially for ^1^O_2_* and ^3^C*), _WS_OOA (which may also include contributions from very aged BB particles) appears to be a more important source of aqueous photooxidants due to its dominance at most ambient locations. There are several important uncertainties in our estimated oxidant concentrations. For example, we do not consider contributions from water-insoluble chromophores, which are significant in atmospheric BrC^41^ and likely also form photooxidants. In addition, photooxidant concentrations vary with the extent of dilution of the particle extract and thus will vary with LWC. However, the relationship with LWC changes between dilute (cloud/fog) and concentrated (ALW) conditions, so our dilute aqueous results here cannot be used for ALW conditions, where concentrations are generally higher.^12,47,48^

In this study, we demonstrated that _WS_BBOA_fresh_ is the most light-absorbing WSOA component and is the dominant contributor to water-soluble BrC light absorption in northern California. In contrast, oxygenated organic species represent 48% of the total WSOA mass but only account for a small fraction (∼12%) of the sunlight absorption by WSOA. Linear regression models applied to the photooxidants (·OH, ^1^O_2_*, and ^3^C*) and WSOA factors enabled the determination of oxidant production potentials of individual WSOA factors. _WS_BBOA_fresh_ is the most potent at producing ^1^O_2_*, whereas _WS_BBOA_fresh_ and _WS_OOAs show comparable production potentials for ^3^C*. Using the oxidant production potentials of _WS_BBOAs and _WS_OOAs, we estimate aqueous-phase oxidant concentrations under cloud and fog conditions at dozens of sites in northern hemisphere. Due to the broad dominance of OOAs in the atmosphere, oxygenated organic species are likely a major contributor to the photooxidants in atmospheric waters.

## Supporting Information Available

Additional details on PM sample collection, locations of wildfires and back trajectory analysis, data comparison between IC, TOC, and AMS measurements, PMF-related calculations, and diagnostics of PMF, and additional figures on AMS analysis of WSOA, PMF results, and contribution of WSOA factors to aqueous-phase oxidants (PDF)
