# Supplementary material for: Chemical and Light-Absorption Properties of Water-Soluble Organic Aerosols in Northern California and Photooxidant Production by Brown Carbon Components
Source: ACS Earth Space Chem. 2023 Apr 24;7(5):1107–19. doi: 10.1021/acsearthspacechem.3c00022 (PMC10202033; doi:10.1021/acsearthspacechem.3c00022)
Supplement: Supplementary file 1 — sp3c00022_si_001.pdf [file sp3c00022_si_001.pdf]

Supporting Information:

Chemical and Light-Absorption Properties of Water-Soluble Organic Aerosols in Northern California and Photooxidant Production by Brown Carbon Components

Wenqing Jiang[1,2], Lan Ma[2,3], Christopher Niedek[1,2], Cort Anastasio[2,3], Qi Zhang[1,2]*

[1]Department of Environmental Toxicology, University of California, 1 Shields Ave., Davis, CA 95616, USA

[2]Agricultural and Environmental Chemistry Graduate Program, University of California, 1 Shields Ave., Davis, CA 95616, USA

[3]Department of Land, Air, and Water Resources, University of California, 1 Shields Ave., Davis, CA 95616, USA

*Corresponding author: Qi Zhang

Email: dkwzhang@ucdavis.edu

Phone: 530-752-5779

Table of Contents

S1. Calculation of species concentration and mass absorption coefficient of PMF factors (p3)

Table S1. Information of PM2.5 sample collection (p4)

Figure S1. Comparison between IC and AMS measurements (p5)

Figure S2. Comparison between TOC and AMS measurements (p6)

Figure S3. Summary of diagnostic plots for the five-factor solution of the PMF analysis (p7)

Figure S4. Correlation between NO_x^+ (or SO_x^+) ions and the total nitrate (or sulfate) signal (p8)

Figure S5. Kolmogorov-Smirnov test results to the samples are representative. (p9)

Figure S6. f44 vs. f43, f44 vs. f60 and Van Krevelen diagram for the WSOA (p10)

Figure S7. Map of the Davis and the adjacent wildfires and back trajectory analysis (p11)

Figure S8. Contributions of the WSOA factors to high m/z AMS signals (p12)

Figure S9. Contributions of the WSOA factors to individual AMS ions (p13)

Figure S10. Correlations between WSOA factors and selected AMS tracer ions (p14)

Figure S11. Contributions of the WSOA factors to oxidant concentrations in PM extracts (p15)

Figure S12. Correlations between oxidant concentration and AMS spectral signals of WSOA (p16)

S1. Calculation of species concentration and mass absorption coefficient of PMF factors

The organic concentration of a PMF factor in solution ($[Org]_i$, μg mL^{-1}) was calculated as:

$$[Org]_i = f_{org,\,i} \times [Total\ Signal]_i \qquad \text{(Eq. S1)}$$

where $f_{org,i}$ is the fraction of organic signals in the factor's spectral profile, and $[Total\ Signal]_i$ is the total signal of the factor. The concentrations of nitrate and sulfate associated with a PMF factor ($[NO3]_i$ and $[SO4]_i$, μg mL^{-1}) in solution were calculated as:

$$[NO3]_i = \frac{f_{NO_x^+,\,i} \times [Total\ Signal]_i}{R_{NO_x^+/HRNO3}} \qquad \text{(Eq. S2)}$$

and

$$[SO4]_i = \frac{f_{SO_x^+,i} \times [Total\ Signal]_i}{R_{SO_x^+/HRSO4}} \qquad \text{(Eq. S3)}$$

where $f_{NOx+,i}$ is the fraction of NO_x^+ ion signals in the factor's spectral profile, $R_{NOx+/HRNO3}$ is the ratio of the NO_x^+ ions to the total nitrate signal (Figure S4a), $f_{SOx+,i}$ is the fraction of SO_x^+ ion signals in the factor's spectral profile, and $R_{SOx+/HRSO4}$ is the ratio of the SO_x^+ ions to the total sulfate signal (Figure S4b). In addition, the species ambient concentrations can be calculated from the concentrations in solution using Eq. 6. The mass absorption coefficient of a PMF factor ($MAC_{\lambda,i}$, m^2 g^{-1}) was calculated as:

$$MAC_{\lambda,\,i} = 2.303 \times \frac{f_{abs,\,\lambda,\,i} \times [Total\ Signal]_i}{[Org]_i} \times 100 \qquad \text{(Eq. S4)}$$

where $f_{abs,\lambda,i}$ is the fraction of the light absorption signal at wavelength λ in the factor's spectral profile, 2.303 is a conversion factor between log10 and natural log, and 100 is for unit conversion.

Table S1. Information of $PM_{2.5}$ sample collection [a]

Sample ID [b]	Sampling Time (hr) [g]	Average $PM_{2.5}$ Concentration ($\mu g\ m^{-3}$) [h]	Estimated Equivalent Liquid Water Content ($mg\ m^{-3}$) [i]
11/12/2019	168 (a week)	13.2	9.4
12/3/2019	24	10.6	65.7
12/17/2019	168 (a week)	9.0	9.4
1/2/2020	24	10.2	65.7
1/3/2020	168 (a week)	10.0	9.4
2/20/2020	24	9.0	65.7
3/4/2020	24	8.4	65.7
7/7/2020	24	7.0	65.7
8/4/2020	24	7.2	65.7
8/19/2020 [c]	24	67.9	65.7
8/24/2020 [c]	24	57.2	65.7
9/9/2020 [c]	24	44.6	65.7
9/15/2020	24	19.5	65.7
10/8/2020	24	33.9	65.7
10/15/2020	24	7.9	65.7
Summer [d, f]	28.8 (average)	49.3	38.3
Winter [e, f]	169.7(average)	9.1	6.6

a. Details of sampling are described in Ma et al.[1]

b. Sample ID represents the first day of sampling for each sample.

c. Samples influenced by wildfires (See fire map and back trajectory analysis in Figure S6).

d. A summer sample that evenly combines filters collected on 8/20/2020 (collected for 24 h), 8/21/2020 (40 hr), and 8/23/2020 (23 hr).

e. A winter sample that evenly combines filters collected on 2/5/2020 (collected for a week), 2/13/2020 (a week), and 2/21/2020 (a week).

f. Samples extracted with 0.7 mL of water per filter square while other samples were extracted with 1 mL of water per filter square.

g. The air flow rate through each 10×8 inch filter was 40 cfm during sampling.

h. Average $PM_{2.5}$ concentration during each sampling period measured at the UC Davis sampling site by the California Air Resources Board.

i. The equivalent liquid water content (LWC, $mg\ m^{-3}$) of each PM extract was calculated as: $LWC = \frac{V_{water} \times \rho_{water}}{T \times Q \times \frac{A_{cut}}{A_{filter}}}$. In this equation, V_{water} is the volume (mL) of water used to extract each square cut of filter, ρ_{water} is the density ($mg\ mL^{-1}$) of water, T is the sampling duration (hr) of the filter, Q is the air flow rate ($m^3\ hr^{-1}$) through the filter during sampling, A_{cut} is the area (cm^2) of the extracted filter square, and A_{filter} is the area (cm^2) of the whole filter.

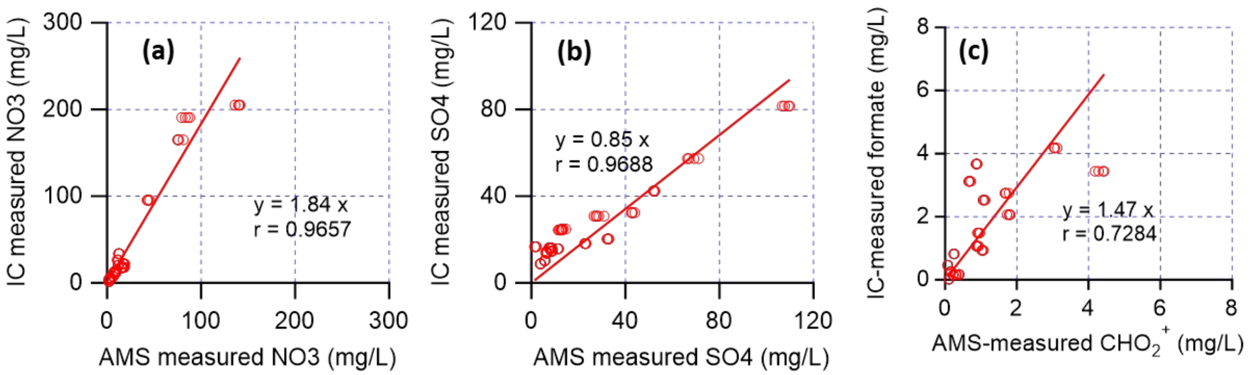

Figure S1. Scatter plots of IC- vs. AMS-measured (a) nitrate and (b) sulfate, and (c) scatter plot of IC-measured formate versus AMS CHO_2^+ in the PM extracts.

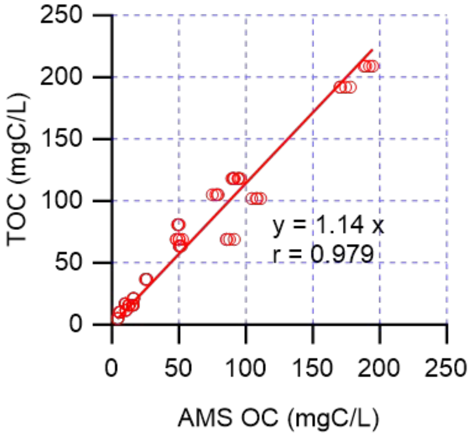

Figure S2. Scatter plots of the total organic carbon concentration measured by TOC analyzer vs. that by AMS.

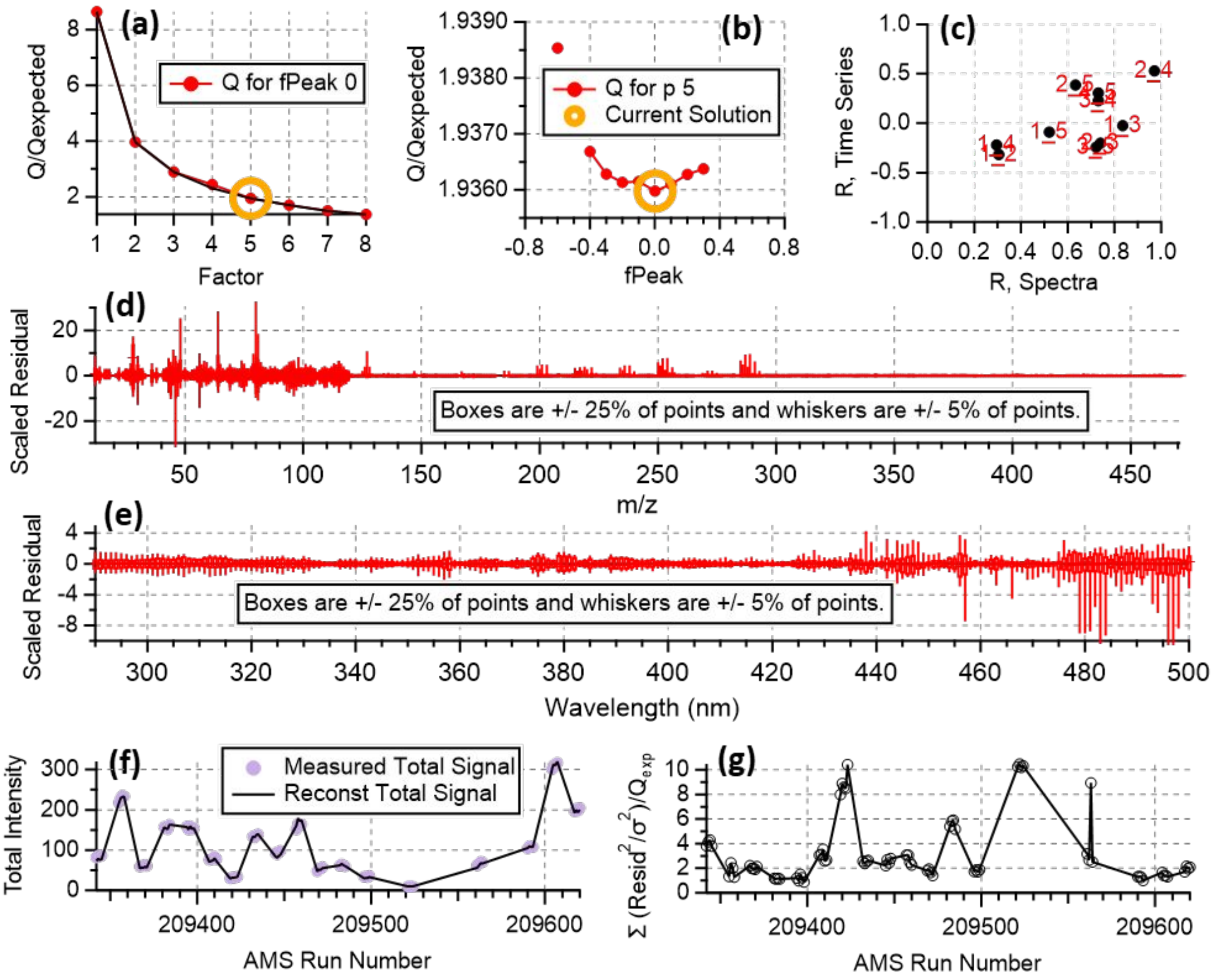

Figure S3. Summary of diagnostic plots for the five-factor solution for PMF analysis: (a) Q/Qexp as a function of number of factors selected for PMF modeling. (b) Q/Qexp as a function of fPeak. (c) Correlations among PMF factors. (d) Box and whisker plot showing the distributions of scaled residuals for each AMS ion. (e) Box and whisker plot showing the distributions of scaled residuals for each light absorption wavelength. (f) Reconstructed and measured total signal for each sample. (g) Q/Qexp for each sample.

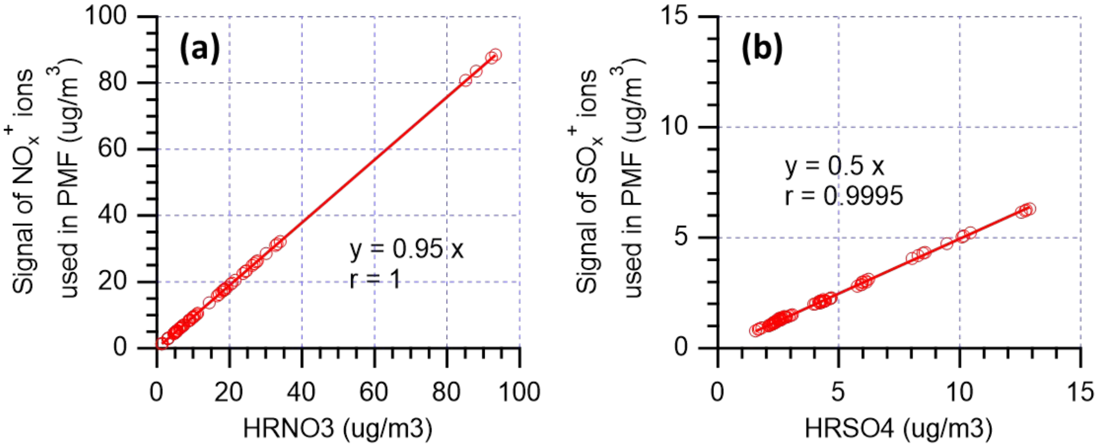

Figure S4. (a) Relationship between the signal of the NO_x^+ ions used in PMF (i.e., NO^+ and NO_2^+) and the total nitrate signal. (b) Relationship between the signal of the SO_x^+ ions used in PMF (i.e., SO^+, SO_2^+, HSO_2^+, SO_3^+, HSO_3^+, $H_2SO_4^+$) and the total sulfate signal.

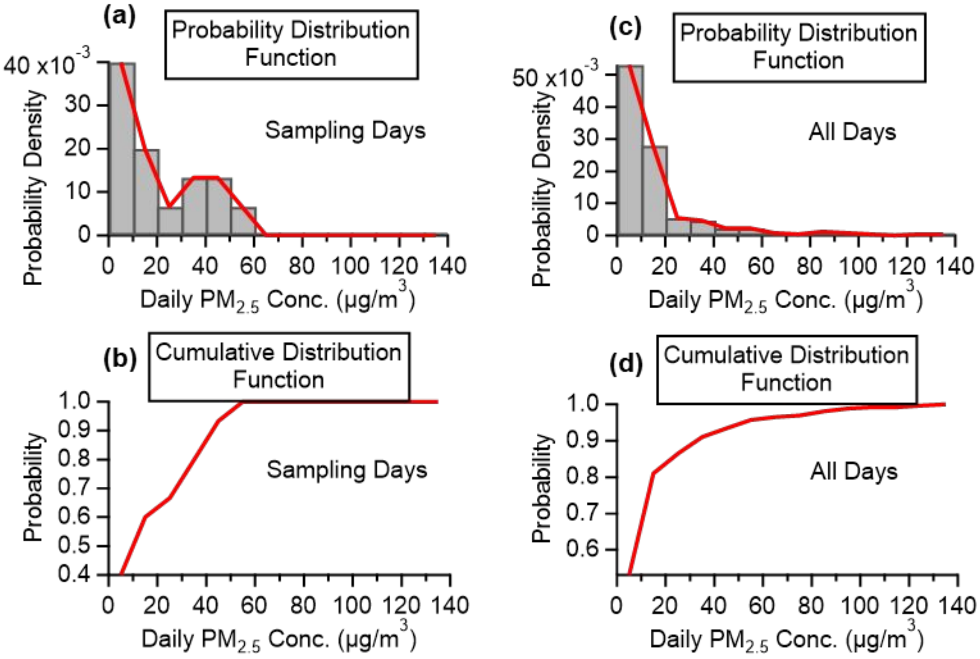

Results of Kolmogorov-Smirnov Test:

alpha	0.05
D	0.29
Critical	0.38
P-Value(Ne)	0.16
P-Value(Marsaglia)	0.17
Critical (B&T)	0.34
P-Value (B&T)	0.15

The test statistic D is lower than the critical value, suggesting the distribution of the daily $PM_{2.5}$ concentration of the sampling days is not significantly different from the distribution of daily $PM_{2.5}$ concentration in all days in 11/2019-10/2020. It suggests that the $PM_{2.5}$ filter samples we collected are representative.

Figure S5. Statistics of the daily ambient $PM_{2.5}$ concentration measured at UC Davis site by California Air Resources and reported on the iADAM online database: (a) probability distribution function (PDF) and (b) cumulative distribution function (CDF) of the daily $PM_{2.5}$ concentration in sampling days. (c) PDF and (d) CDF of the daily $PM_{2.5}$ concentration in all the days from November 2019 to October 2020. The table shows the results of the Kolmogorov-Smirnov test performed between the daily $PM_{2.5}$ concentration in sampling days and that in all the days from November 2019 to October 2020.

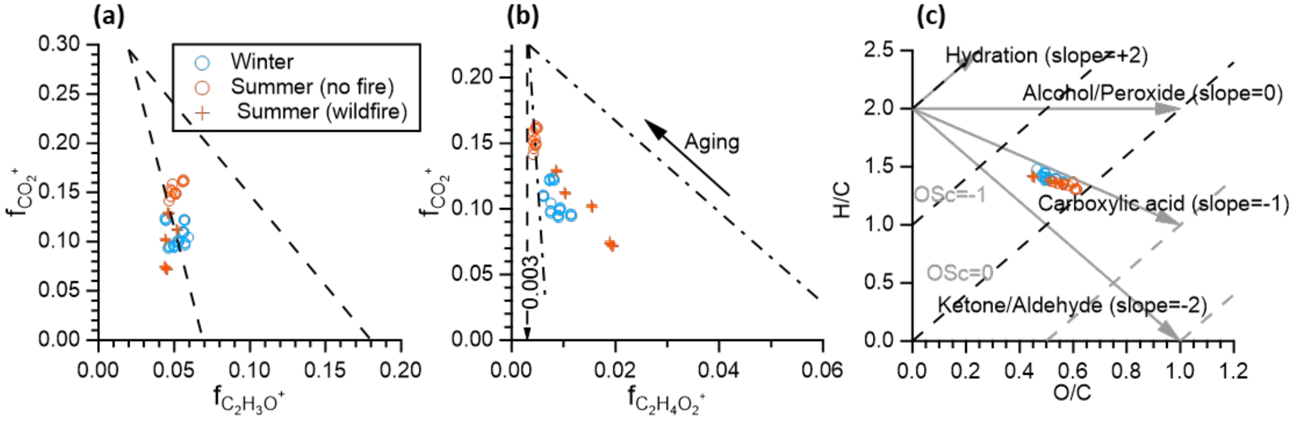

Figure S6. (a) f44 vs. f43[2], (b) f44 vs. f60[3] and (c) Van Krevelen diagram[4] for the WSOA in the PM extracts.

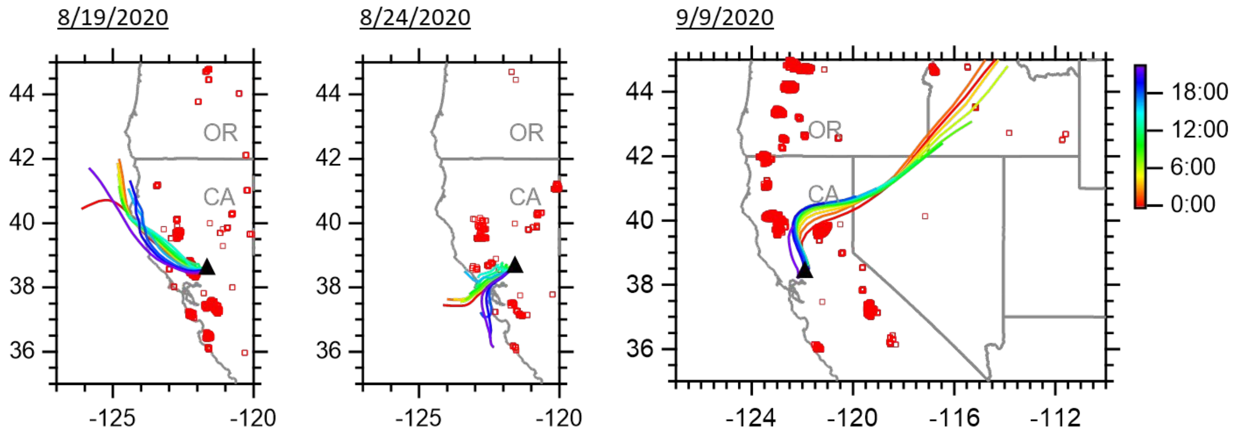

Figure S7. Back trajectories for the three 24-hour samples (8/19/2020, 8/24/2020, and 9/9/2020) most strongly influenced by wildfires. The black triangle is the sample collection site of Davis, while red squares represent fires detected by the MODIS satellite and lines represent 24-hour HYSPLIT back trajectories of air masses arriving from 02:00 to 24:00 (PST) every 2 h on the collection day.

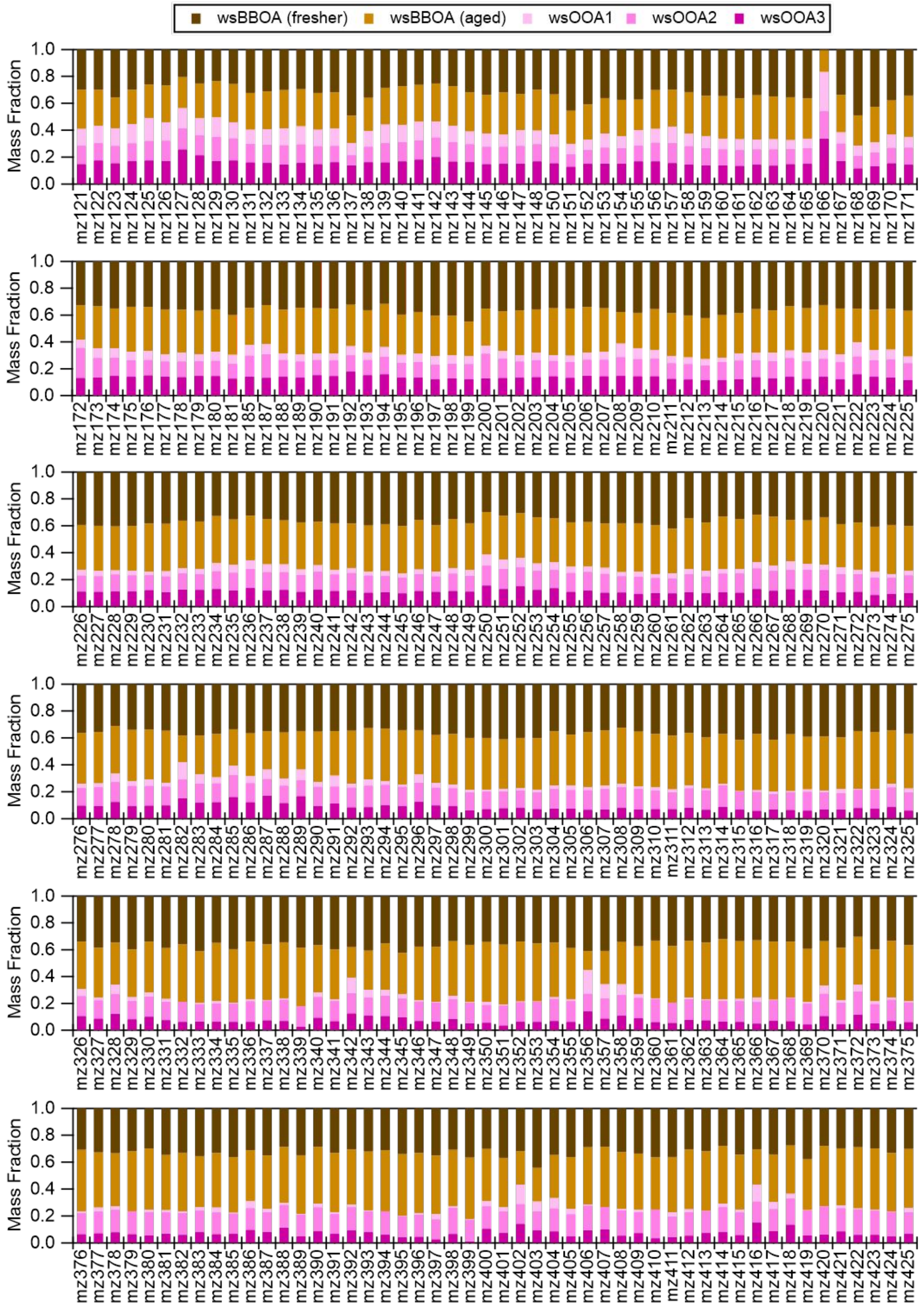

Figure S8. Contributions of the five WSOA factors to the high m/z ions (m/z > 120, unit mass resolution) in the PM extracts.

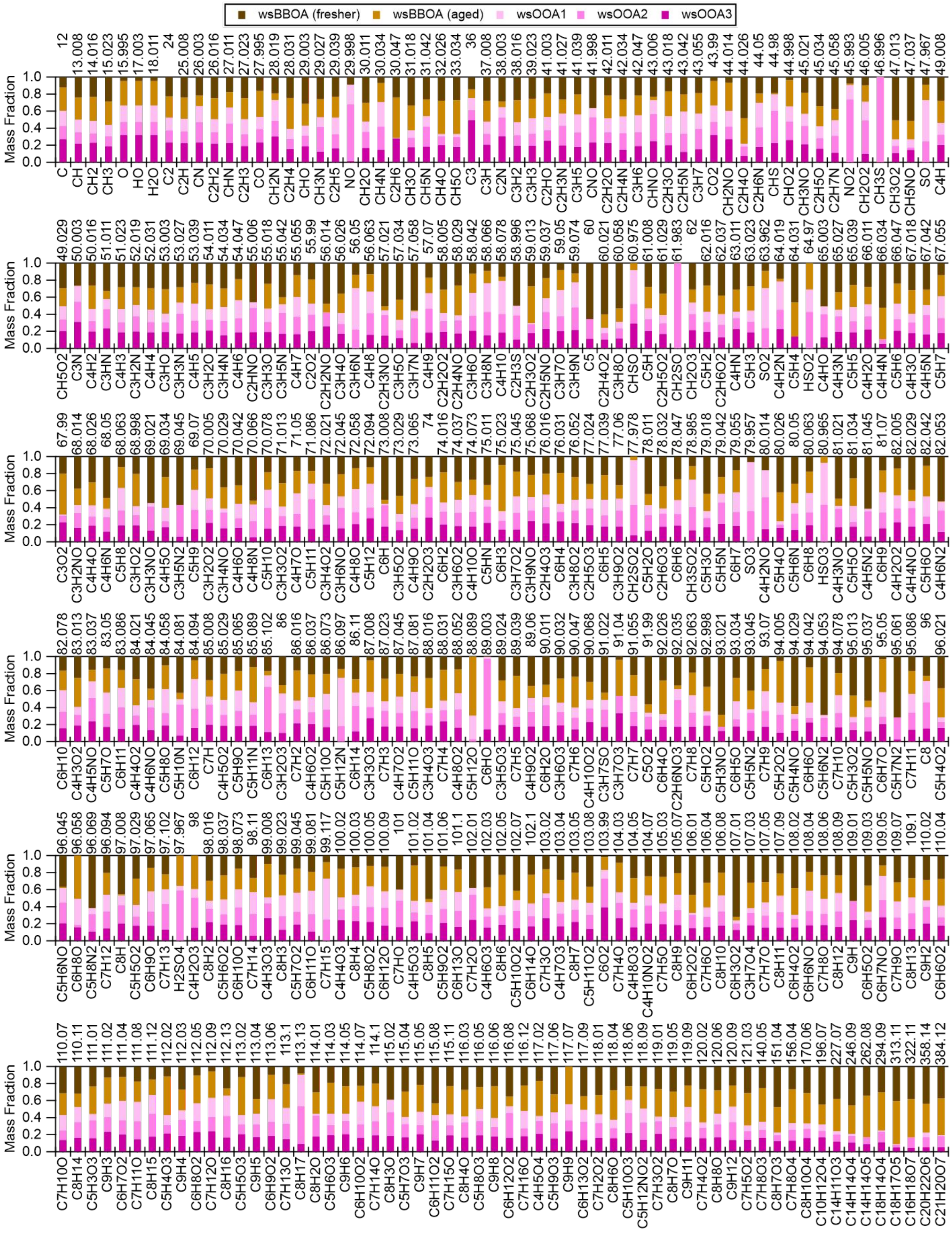

Figure S9. Contributions of the five WSOA factors to individual AMS ions in the PM extracts.

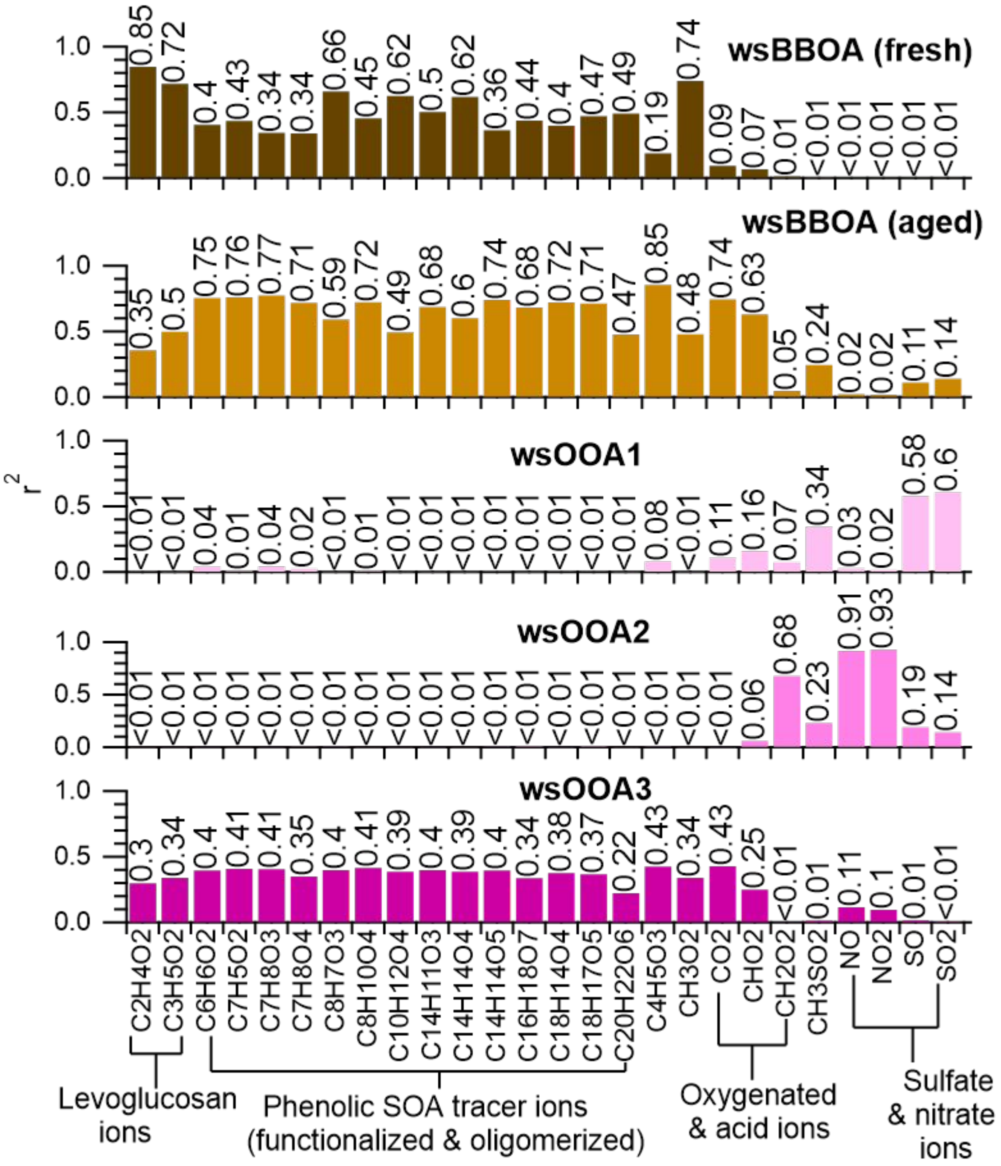

Figure S10. Correlations between WSOA factors and selected tracer ions, including fresh BBOA tracers[3], phenolic tracers[5,6], oxygenated organic tracers, and sulfate and nitrate tracers.

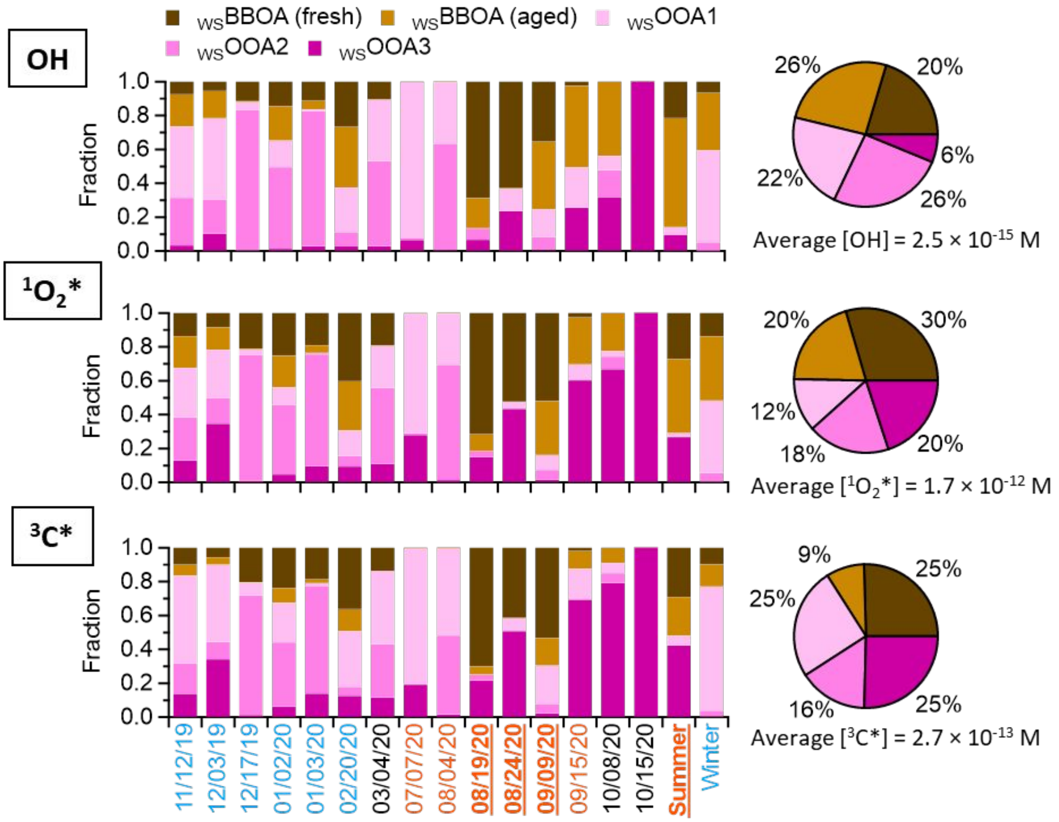

Figure S11. Fractional contributions of the five WSOA factors to oxidant (•OH, $^1O_2^*$ and $^3C^*$) concentrations in PM extracts.

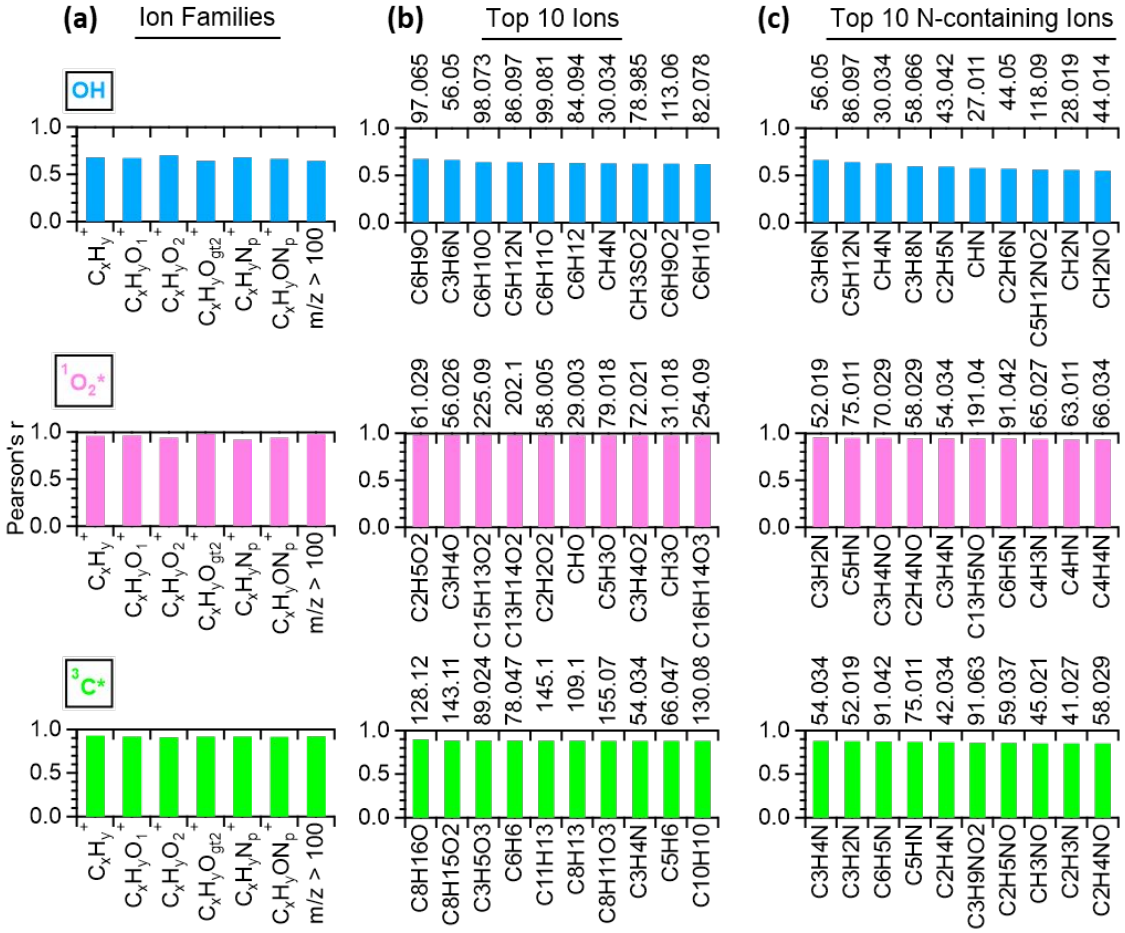

Figure S12. Pearson's r values for the correlations between concentrations of aqueous-phase oxidants (OH, $^1O_2$*, or ^3C*) measured in illuminated $PM_{2.5}$ extracts and AMS spectral signals for (a) WSOA ion families; (b) 10 best correlated WSOA ions; and (c) 10 best correlated N-containing WSOA ions. In (b) and (c), the bottom and top axes denote the elemental compositions and the exact masses of the corresponding ions.

Reference

(1) Ma, L.; Worland, R.; Heinlein, L.; Guzman, C.; Jiang, W.; Niedek, C.; Bein, K.; Zhang, Q.; Anastasio, C. Seasonal Variation in Light Absorption and Photooxidant Formation in Aqueous Extracts of Ambient Particles. In Preparation.

(2) Ng, N. L.; Canagaratna, M. R.; Zhang, Q.; Jimenez, J. L.; Tian, J.; Ulbrich, I. M.; Kroll, J. H.; Docherty, K. S.; Chhabra, P. S.; Bahreini, R.; et al. Organic Aerosol Components Observed in Northern Hemispheric Datasets from Aerosol Mass Spectrometry. *Atmos. Chem. Phys.* **2010**, *10* (10), 4625–4641. https://doi.org/10.5194/acp-10-4625-2010.

(3) Cubison, M. J.; Ortega, A. M.; Hayes, P. L.; Farmer, D. K.; Day, D.; Lechner, M. J.; Brune, W. H.; Apel, E.; Diskin, G. S.; Fisher, J. A.; et al. Effects of Aging on Organic Aerosol from Open Biomass Burning Smoke in Aircraft and Laboratory Studies. *Atmos. Chem. Phys.* **2011**, *11* (23), 12049–12064. https://doi.org/10.5194/acp-11-12049-2011.

(4) Heald, C. L.; Kroll, J. H.; Jimenez, J. L.; Docherty, K. S.; DeCarlo, P. F.; Aiken, A. C.; Chen, Q.; Martin, S. T.; Farmer, D. K.; Artaxo, P. A Simplified Description of the Evolution of Organic Aerosol Composition in the Atmosphere. *Geophys. Res. Lett.* **2010**, *37* (8), L08803. https://doi.org/10.1029/2010GL042737.

(5) Yu, L.; Smith, J.; Laskin, A.; Anastasio, C.; Laskin, J.; Zhang, Q. Chemical Characterization of SOA Formed from Aqueous-Phase Reactions of Phenols with the Triplet Excited State of Carbonyl and Hydroxyl Radical. *Atmos. Chem. Phys.* **2014**, *14* (24), 13801–13816. https://doi.org/10.5194/acp-14-13801-2014.

(6) Jiang, W.; Misovich, M. V; Hettiyadura, A. P. S.; Laskin, A.; McFall, A. S.; Anastasio, C.; Zhang, Q. Photosensitized Reactions of a Phenolic Carbonyl from Wood Combustion in the Aqueous Phase—Chemical Evolution and Light Absorption Properties of AqSOA. *Environ. Sci. Technol.* **2021**, *55* (8), 5199–5211. https://doi.org/10.1021/acs.est.0c07581.
